# Effectiveness of photodynamic therapy for mammary and extra-mammary Paget's disease: a state of the science review

**DOI:** 10.1186/1471-5945-11-13

**Published:** 2011-06-15

**Authors:** Alexa A Nardelli, Tania Stafinski, Devidas Menon

**Affiliations:** 1 Department of Public Health Sciences, University of Alberta, Room 3021, Research Transition Facility, 8308 114 Street, Edmonton, Alberta, T6G 2V2, Canada

## Abstract

**Background:**

Paget's disease is a rare skin disorder occurring in the breast (mammary) or in the groin, genital, peri-anal and axillary regions (extra-mammary). Typical treatment involves surgical excision, which in the case of extra-mammary Paget's disease, can lead to significant morbidity. Photodynamic therapy (PDT) which uses a topical or intravenous photosensitizing agent that is activated by a light source to ablate abnormal tissue, offers a minimally invasive alternative. The purpose of this study was to assess the effectiveness of photodynamic therapy in the treatment of Paget's disease.

**Methods:**

Following Cochrane guidelines, a comprehensive systematic review of all clinical studies and reports examining the use of PDT for mammary and extra-mammary Paget's disease was conducted. Study quality was assessed using the Oxford Levels of Evidence Scale.

**Results:**

21 retrospective and 2 prospective non-comparative studies were identified and included in the review: 9 case reports with 1-2 patients and 14 case series with 1-16 patients. These reports totalled 99 patients with 133 extra-mammary Paget's lesions and 3 patients (with 3 lesions) with mammary Paget's disease. Follow-up periods were typically one year or less, with 77/133 extra-mammary lesions exhibiting complete response to PDT. One recurrent mammary skin lesion and two mammary lesions treated concomitantly with surgery also exhibited complete responses.

**Conclusions:**

Evidence of the effectiveness of PDT for Paget's disease is promising, but limited. This may, in part, be explained by the rarity of the condition, making controlled comparative clinical trials challenging.

## Background

Paget's disease is an extremely rare form of intraepithelial adenocarcinoma that may have an underlying tumour component. It primarily affects Caucasian women over the age of 50[1]. Since in its early stages symptoms are often minor, individuals may not seek medical attention for several years, delaying diagnosis and treatment[1-3]. In addition, the condition can be misdiagnosed as eczema or dermatitis,[4] with a benign dermatologic diagnosis creating further delays[5].

Paget's disease is categorized as either mammary or extra-mammary. While the two types are histologically the same (the epidermis shows diffuse infiltration of large vacuolated cells with a bluish cytoplasm (Paget cells)), the location differs[6]. Mammary Paget's disease involves the skin of the breast, in or around the nipple. Most individuals with the condition (> 92%) have underlying breast cancer (either ductal carcinoma in situ or invasive breast cancer)[2,7]. Extra-mammary Paget's disease mainly affects the perianal and genital areas, or elsewhere on the skin near apocrine glands,[6] and is extremely rare (only a few hundred reports have been documented in peer-reviewed literature)[6]. In many patients, the disease may exist for 10 to 15 years without progressing. Unlike mammary Paget's, a smaller proportion of cases exhibit an underlying neoplastic component (approximately 25% have carcinoma of the Bartholin glands, urethra, bladder, vagina, cervix, endometrium, prostate, rectum, or colon)[6].

Treatment typically involves surgical excision. However, this can lead to significant morbidity, especially in the case of extra-mammary Paget's disease, where the lesions are often large. Additionally, the disease primarily affects older populations, not all of whom can tolerate surgery. Thus, given the potential slow progression of the disease, considerable interest in less invasive approaches exists[8]. One such approach is photodynamic therapy (PDT). PDT uses a photosensitizing agent that, when activated by light of a particular wavelength, induces a chemical reaction within the cells, destroying the affected tissue[9,10]. Since the photosensitizing agent is more readily absorbed by abnormal cells, healthy tissue is spared. While topical photosensitizing agents are most commonly used (Levulan^® ^Kerastick^® ^(a type of 5-aminolevulinic acid or ALA) and Metvix^® ^(a type of methyl aminolevulinate or MAL)), intravenously administered versions (hematoporphyrin derivatives, such as porfimer sodium) are also available[11].

The purpose of this project was to assess the clinical effectiveness of PDT for mammary and extra-mammary Paget's disease, based on existing published, peer-reviewed clinical studies.

## Methods

A systematic review of relevant studies was undertaken following Cochrane guidelines and the QUORUM statement[12].

### Identification of potentially relevant studies

To identify studies published as of February 2011, a structured search strategy, which combined relevant controlled vocabulary terms (Medical Subject Headings (MeSH) in MEDLINE, and EMTREE thesaurus terms in EMBASE) with additional non-index terms was first developed. Such terms included photochemotherapy, photosensitizing agent(s), photodynamic therapy or PDT, and Paget's disease (both mammary and extra-mammary). The search strategy was then applied to the following electronic bibliographic databases: PubMed (MEDLINE and non-MEDLINE), The Cochrane Library, EMBASE, the UK Centre for Reviews and Dissemination databases (DARE, HTA and NHS EED), CINAHL, PsycINFO, and Web of Science. No date, language or other limits were applied. Grey literature was also searched, including guidelines and clinical trials web sites, conference proceedings, Google and checking the reference lists of relevant articles. Full search details are provided in Additional file 1.

### Selection of studies for inclusion in the review

Two researchers independently screened the titles and abstracts of citations identified through the literature search using predetermined inclusion criteria (Table 1). Corresponding papers of those deemed potentially relevant were then retrieved for full review by the same two researchers. Disagreements between them were resolved through discussion and, if necessary, third party adjudication.

**Table 1:** PICOS Elements of the Review Protocol

Parameter	Inclusion Criteria	Exclusion Criteria
***P**articipants*	● Studies of participants diagnosed with Paget's disease (mammary or extramammary, invasive or non-invasive) and 18 years of age or older	● Studies of participants under 18 years of age

***I**ntervention*	● Any application of a photodynamic therapy alone or in conjunction with another therapy ● All wavelengths and light sources will be considered ● Any formulation of ALA, including experimental formulations ● MAL ● All intravenous photosensitizers	● None

***C**omparator*	● All studies with or without comparators	

***O**utcomes*	● Any clinical outcome, including (but not restricted to): complete lesion eradication; cosmetic results; patient satisfaction; adverse effects and the need for additional therapy	● Studies without any defined clinical outcomes

***S**tudy Design*	● Any study design including case series and case reports	● Review articles ● Economic evaluations ● Editorials and opinion pieces

### Extraction of data from included studies

Information from included studies was systematically extracted by two independent reviewers using a pre-tested data abstraction form, accompanied by a set of decision rules. The abstraction form included elements related to study setting, sample size, and design; comparator (where applicable); outcomes measured (both cosmetic and clinical); and findings. Reviewers compared results and discrepancies were, once again, resolved through discussion and, if necessary, third party adjudication.

### Critical appraisal of included studies

Studies were appraised using the Oxford Centre for Evidence-based Medicine Levels of Evidence and Grades of Recommendation[13].

### Data analysis and synthesis of results

Data extracted from studies were summarized in tabular form to facilitate qualitative analyses of trends or patterns in the findings. Where possible, weighted pooled means for outcomes, such as percentage of patients who experienced complete eradication of lesions were also calculated in order to generate summary point estimates.

## Results

**Figure 1:**
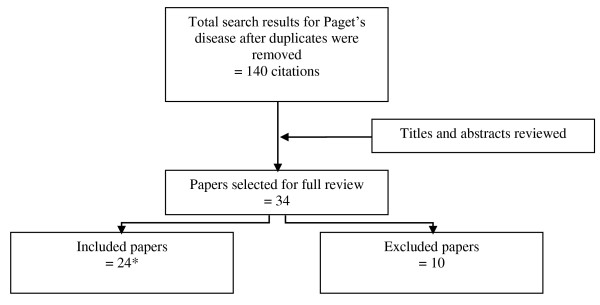
**Literature search results and study selection for clinical review of Paget's Disease**. *Two papers had reports of duplicate patient data resulting in 24 papers and 23 included studies overall.

Results of the literature search are shown in Figure 1. Of 140 papers initially identified (after removing duplicates), 34 Paget's papers were retrieved for full review and 32 initially met the inclusion criteria. Two of the 32 papers that met the inclusion criteria were excluded because appropriate outcomes could not be obtained: 1 study was a cohort of patients with skin lesions who were treated with PDT that included one patient with EMPD whose individual treatment outcomes could not be determined from the report[5] and the other study was a review of cases in a vulval Paget's disease patient registry that included two patients treated with PDT whose individual treatment outcomes could not be determined from the report[14]. Six of the 32 papers that met the inclusion criteria were further excluded because patients had already been included in other studies. Additionally, two papers reported duplicate data, resulting in 24 papers of 23 studies included in the final review.

### Description of included studies

The 23 studies comprised single-centred case reports of 1-2 patients (9) and case series of 1-16 patients (14) carried out in China (7), Japan (4), Korea (3), UK (2), US (2), Austria (1), Italy (1), Belgium (1), Israel (1), and Brazil (1) (Table 2). Collectively, they involved 102 patients (34 female, 51 male, 17 unspecified gender), ranging in age from 28 to 92 years (majority over 50 years), and 136 lesions treated with PDT. Of the 102 patients, the majority had extra-mammary Paget's disease (88). Among the remaining 14, 3 had mammary Paget's disease and 11 were unspecified. Most studies excluded invasive or metastasized EMPD; 3 patients in 3 studies had an underlying carcinoma and/or lymphatic involvement. Only a few studies reported the Fitzpatrick skin type of included patients: in 1 study, all 16 patients were Fitzpatrick skin type IV or V (Asian)[15] and in another, 7 of 8 patients included were skin type I or II and 1 patient was type III[11,16]. Four studies reported the race of patients, 3 studies with 12 patients, all Caucasian[1,17,18] and a case report on one Chinese patient[3]. In patients with extra-mammary Paget's disease, the majority of lesions appeared in the groin/pubis/genitalia, regardless of gender (female: 28 lesions; male: 47 lesions) and in the perianal region in females (15 lesions). Across all lesion types, approximately one third (44/136) had received prior treatment (including mainly surgical and/or laser excision (37); topical ALA-PDT (4), cryotherapy (1), topical imiquimod (1), and irradiation and chemotherapy (1)). Heterogeneity in PDT treatment regimens between studies existed, however PDT was typically performed with topical 5-ALA or m-ALA, or intravenous hematoporphyrin derivatives activated by red light either in the form of visible light or laser. The number of PDT treatments per lesion ranged from1 to 10, with the majority of lesions receiving 1-3 treatments. Descriptions of each study are presented in Additional file 2.

**Table 2:** Characteristics of included studies

Study	Country	Diagnosis	Age range (years)	No. of patients	**No. of lesions**^a^
**Prospective case series**					

Li et al. (2010)[15]	China	EMPD	54-83	16	21
Raspagliesi et al. (2006)[17]	Italy	EMPD	55-75	7	11

**Retrospective case reports/case series**					

Housel et al. (2010)[16] & Shieh et al. (2002)[11]	USA	EMPD	50-75	8	24
Thaler et al. (2010)[25]	Austria	EMPD	69	1	1
Andretta-Tanaka et al. (2009)[1]	Brazil	EMPD	67-76	4	4
Fukui et al. (2009)[44]	Japan	EMPD	66-84	5	8
Li et al. (2009)[3]	China	EMPD	75	1	1
Wang et al. (2008)[26]	China	EMPD MPD	66^b^ 28^b^	3 1	NR^a^ NR^a^
Liu et al. (2007)[4]	China	EMPD	64-80	5	5
T'kint & Roseeuw (2006)[45]	Belgium	EMPD	64	1	2
Kim et al. (2005)[46]	Korea	EMPD	54-73	7	NR^a^
Madan et al.(2005)[21]	UK	EMPD	80	1	1
Mikasa et al. (2005)[47]	Japan	EMPD	73-92	2	NR^a^
Tulchinsky et al. (2004)[8]	Israel	EMPD	49-74	2	NR^a^
Zawislak et al. (2004)[18]	UK	EMPD	66	1	1
Zhu et al. (2004)[48]	China	PD^c^	NR	8	8
Song et al. (2003)[49]	Korea	EMPD	51-78	2	6
Xu et al. (2002)[19]	China	EMPD MPD	50-84	8 2	8 2
Chang et al. (2001)[50]	Korea	EMPD	50-73	7	8
Runfola et al. (2000)[20]	USA	EMPD	79	1	1
Henta et al. (1999)[22]	Japan	EMPD	74	1	1
Wang et al. (1991)[23]	China	EMPD PD^c^	NR NR	4 3	4 3
Kubota et al. (1986)[51]	Japan	EMPD	77	1	1

**Total: 23 studies**			**28-92**	**102**	**136**^a^

^a ^total no. of lesions includes all lesions entering study; if a study did not report number of lesions it was assumed that each patient had 1 lesion

^b ^age only reported for 2 of 4 patients

^c ^unspecified Paget's disease

EMPD = extra-mammary Paget's disease, MPD = mammary Paget's disease, NR = not reported

### Quality of included studies

Based on the Oxford Centre for Evidence-based Medicine Levels of Evidence, the quality of the studies was low. All except 2 were retrospective and all were non-comparative and small in sample size. In general, study design was attributed to the rarity of the condition.

### Safety

Half of the included studies reported adverse events. Across these studies, adverse events appeared mild and transient and included mainly local pruritus, erythema, swelling, blistering, effusion, superficial erosions, crust formation and/or peeling[4,15-17,19-21]. Local phototoxicity/photosensitivity was also reported in all patients in 2 studies (n = 7 and n = 1)[17,21]. Treatment-related pain was commonly experienced[15,17,18,20,22] and in 1 prospective study (16 patients) ranged from 2-10 (mean ± SD: 5.4 ± 1.3) on a VAS scale (0-none, 10-extreme), with patients with perianal and vulval lesions rating pain higher than those with axillary or scrotal lesions[15]. The single study that assessed liver function post treatment with PDT reported normal values[23].

### Clinical effectiveness

An assessment of clinical effectiveness included response of lesion to treatment (may include lesion recurrence) reported by all 23 studies, and cosmetic or anatomical function-related outcomes, reported by some studies. Individual lesion response outcomes for each study are presented in Additional files 3 and 4.

### Extra-mammary Paget's disease (EMPD)

#### Lesion response

In 99 patients with a total of 133 extra-mammary lesions, 77 of the lesions showed a complete response, 52 a partial response, and 4 a minimal or no response to PDT. No difference in response patterns between previously treated lesions and those that had received no prior treatment was apparent.

Two of the 23 studies included were prospective case series. One prospective study of 16 patients with 21 lesions reported a 66% (14/21) clinical complete response rate after 3 topical ALA-PDT treatments and a 50% recurrence rate, dropping the complete response rate to 33% (7/21) at 24 months[15]. The other prospective study of 7 patients with 11 lesions found a 75% complete response rate 1-5 months after 3 topical MAL-PDT treatments and did not report any longer term outcomes[17].

Of 76 patients with 101 lesions from retrospective studies, 62 lesions demonstrated a complete response, 37 a partial response and 2 minimal to no response to PDT. No differences in response patterns across type of PDT used were evident. Long-term (> 2 year) follow-up of patients was reported in 2 of the 16 retrospective studies[8,11,16,20]. Of 5 lesions (in 4 patients) showing complete responses after treatment with intravenous porfimer sodium-PDT, 4 lesions remained recurrence free 62-96 months after treatment and 1 lesion recurred 48 months after treatment[11,16,20]. A complete response was also maintained (47 and 88 months after treatment) in 2 lesions in a patient treated with topical ALA-PDT[11,16].

#### Cosmetic and/or functional response

Good cosmetic outcomes were demonstrated after PDT in both of the prospective case series[15,17]. In the study of 16 patients, 100% of the patients with a complete response reported satisfaction with their cosmetic outcomes[15]. In this study, investigator rated cosmetic outcome was excellent or good in 36% (5/14) of the lesions with incomplete responses and poor in 64% (9/14) of the lesions. Reasons for a poor cosmetic outcome included depigmentation (4), atrophy (2), redness (2) and induration (1)[15]. An acceptable cosmetic outcome with no scarring and no substantial changes in baseline function or anatomic profile was reported in all patients with a complete response in the other prospective study[17].

In a retrospective case series of 8 patients, excellent cosmetic outcomes with no scarring were reported in the 5 patients treated with ALA-PDT[24]. Scarring was reported in the 3 patients treated with porfimer-sodium PDT, however the scarring was documented as less severe than prior surgical scars[11]. No post-treatment functional impairments were found in any of the 8 patients[11,24]. Several other case reports described preserved anatomical functionality and improvements in patient comfort and quality of life after PDT[1,3,22,25].

### Mammary Paget's disease (MPD)

#### Lesion response

There are only three reports of the use of PDT for MPD. One patient with a recurrent lesion after radical mastectomy showed a complete response after treatment with topical PDT[26]. No recurrence was reported for this patient 12 months after treatment. No treatment history, surgical description, or follow-up period were specified for the other two patients, however both were reported to have a complete responses after combined surgical excision and topical PDT[19].

#### Cosmetic and/or functional response

No evidence was available regarding cosmetic and functional outcomes after PDT for the treatment of MPD.

## Discussion

This review aimed to assess the clinical effectiveness of photodynamic therapy for the treatment of Paget's disease. Based on the limited quantity and quality of studies found, the use of PDT for Paget's disease appears safe and well tolerated. There were no reports of mortality or significant adverse events related to PDT, however, rare complications may not be observed in these small studies. Although a complete response following PDT was achieved in approximately half of the 103 extra-mammary lesions, the durability of this response remains unclear, since follow-up periods were typically less than one year. Nevertheless, good cosmetic and functional outcomes were demonstrated and, in general, patients appeared satisfied with PDT. Evidence of the short and long term effectiveness of PDT for MPD was limited, with only 3 case reports included in the review. While PDT yielded complete responses in all 3 cases, 1 of the 3 had received previous surgical treatment and the other 2 had received PDT combined with surgery.

It has been suggested that the effectiveness of PDT may depend on several factors, such as lesion location, presence or absence of underlying carcinomas and patient skin type[1,7,15,27,28]. In the studies included in this review, no trends associated with these factors emerged.

The standard treatment for EMPD is surgical excision, which is often extensive, involving large margins and reconstructive surgery. Also, it is frequently associated with anatomical and functional impairments[7,17,29]. Histologically, Paget's disease typically extends beyond the visible lesion present, making it difficult to obtain a complete excision[7,29]. Consequently, high recurrence rates after surgery for EMPD have been shown (studies report approximately 30 to 70% of lesions recur after treatment)[1,7,27,30-32]. Patients with in-situ EMPD are less likely to show recurrences (~35% of lesions) in comparison to patients with invasive EMPD (up to 67% of lesions)[27,33]. Although no comparative studies of PDT were found, other treatment options exist. For example, Moh's micrographic surgery has been associated with lower recurrence rates (8-28%), as well as less morbidity compared to standard surgical excision[1,30-32,34]. Laser therapy, radiotherapy and/or systemic chemotherapy, topical pharmacotherapies (imiquimod or 5-FU) and cryotherapy have also been used. Recent studies of carbon dioxide (CO_2_) laser treatment of EMPD lesions report recurrence rates similar to those following surgical excision (~30-70%) but also significant pain[27,35]. Radiotherapy can be used as a primary treatment or as an adjuvant treatment after surgical excision[36]. Recurrence rates from 0 to 60% have been reported, with no apparent differences between use as a primary treatment or as a postoperative adjuvant[37-39]. Systemic chemotherapy applied in combination with radiotherapy appears to improve responses and prevent recurrences, but the use of systemic chemotherapy, alone, requires further investigation[27]. Comparable response rates with no subsequent recurrences after the use of imiquimod cream for the treatment of non-invasive EMPD in a small number of cases with short follow-up periods have been demonstrated[1,40]. However, in a larger case series, treatment of EMPD with imiquimod resulted in poor response rates (38%)[39]. Little evidence on the use of cryotherapy to treat EMPD exists; therefore, it is unclear whether it offers a suitable primary treatment option.

The standard treatment for MPD (regardless of the presence or absence of underlying breast cancer) is a mastectomy with or without lymph node removal[41]. Mastectomy alone (regardless of lymphatic removal) for the treatment of in situ lesions has demonstrated 5-year recurrence free survival rates of 90% however, recurrence free survival drops to 63% in patients with underlying invasive carcinomas[42]. Adjuvant chemotherapy and/or radiation may be used to decrease recurrences[41]. Other more breast conserving treatment options include excision of the nipple, lumpectomy and radiation alone however, when used to treat patients with underlying breast cancer, these approaches may be associated with high recurrence rates[2,41]. Local excision combined with radiation has been shown to be a highly effective treatment for MPD without underlying breast cancer with high rates of long-term disease-free survival, showing 11% recurrence rates in a long-term, collaborative, multi-centre study[43].

With the limited amount and low level of evidence available, definitive conclusions on the use of PDT for the treatment of Paget's disease are not possible. However, given the significant morbidity associated with surgery and the frequency of recurrence associated with alternate treatment options, PDT for primary or recurrent EMPD appears promising and warrants further comparative investigation. In the rare case of MPD without underlying breast cancer, PDT may offer a breast-conserving treatment option or an adjuvant to surgery. While given the rarity of this disease, randomized controlled trials may not be feasible, comparative data could be collected through a prospective, international registry.

## Conclusions

No evidence on the comparative effectiveness of PDT for the treatment of Paget's disease is available. However, existing non-comparative studies suggest PDT offers a promising less-invasive alternative worthy of further investigation as a primary or adjuvant treatment option, especially given the significant morbidity associated with the standard treatment option (surgical excision).

## Competing interests

The authors declare that they have no competing interests.

## Authors' contributions

AN was involved in the acquisition, analysis and interpretation of the data, and drafting and revising the manuscript. TS made a substantial contribution to the conception and design of the study, interpretation of data, and was involved in writing and critically reviewing drafts of the manuscript. DM made substantial contributions to the conception and design of the study, to interpretation of the data, and has reviewed the manuscript. All authors have given approval for submission of this version.

## Pre-publication history

The pre-publication history for this paper can be accessed here:

http://www.biomedcentral.com/1471-5945/11/13/prepub

## Supplementary Material

Additional file 1 **Literature search**. Details of the literature search strategy and search results are provided in additional file 1. Click here for file

Additional file 2 **Table of included studies of photodynamic therapy (PDT) for Paget's disease**. Details of study and patient characteristics, interventions, outcomes and quality of included studies are provided in additional file 2. Click here for file

Additional file 3 **Studies of photodynamic therapy (PDT) for Extramammary Paget's disease: lesion response**. Details of individual patient and lesion characteristics, type and number of PDT treatments, lesion response outcomes and lengths of follow-up are provided in additional file 3. Click here for file

Additional file 4 **Studies of photodynamic therapy (PDT) for Mammary Paget's disease: lesion response**. Details of individual patient and lesion characteristics, type and number of PDT treatments, lesion response outcomes and lengths of follow-up are provided in additional file 4. Click here for file
