# Supplementary material for: Effectiveness of photodynamic therapy for mammary and extra-mammary Paget's disease: a state of the science review
Source: BMC Dermatol. 2011 Jun 15;11:13. doi: 10.1186/1471-5945-11-13 (PMC3141658; doi:10.1186/1471-5945-11-13)
Supplement: Additional file 1 — Literature search. Details of the literature search strategy and search results are provided in additional file 1. [file 1471-5945-11-13-S1.DOCX]

**Additional file 1 - Literature search**

### Database Searches

1. PubMed (February 2, 2011)

| **Search** | | **Result** |
| --- | --- | --- |
| [#21](http://www.ncbi.nlm.nih.gov/pubmed/?querykey=21&dbase=pubmed&querytype=eSearch&) | Search #16 AND #20 | [41](http://www.ncbi.nlm.nih.gov/pubmed/?cmd=HistorySearch&querykey=21&) |
| [#20](http://www.ncbi.nlm.nih.gov/pubmed/?querykey=20&dbase=pubmed&querytype=eSearch&) | Search #17 OR #18 OR #19 | [7347](http://www.ncbi.nlm.nih.gov/pubmed/?cmd=HistorySearch&querykey=20&) |
| [#19](http://www.ncbi.nlm.nih.gov/pubmed/?querykey=19&dbase=pubmed&querytype=eSearch&) | Search paget*[tiab] | [7022](http://www.ncbi.nlm.nih.gov/pubmed/?cmd=HistorySearch&querykey=19&) |
| [#18](http://www.ncbi.nlm.nih.gov/pubmed/?querykey=18&dbase=pubmed&querytype=eSearch&) | Search paget's disease, mammary | [695](http://www.ncbi.nlm.nih.gov/pubmed/?cmd=HistorySearch&querykey=18&) |
| [#17](http://www.ncbi.nlm.nih.gov/pubmed/?querykey=17&dbase=pubmed&querytype=eSearch&) | Search paget disease, extramammary | [1265](http://www.ncbi.nlm.nih.gov/pubmed/?cmd=HistorySearch&querykey=17&) |
| [#16](http://www.ncbi.nlm.nih.gov/pubmed/?querykey=16&dbase=pubmed&querytype=eSearch&) | Search #1 OR #2 OR #3 OR #4 OR #5 OR #6 OR #7 OR #8 OR #9 OR #10 OR #11 OR #12 OR #13 OR #14 OR #15 | [74375](http://www.ncbi.nlm.nih.gov/pubmed/?cmd=HistorySearch&querykey=16&) |
| [#15](http://www.ncbi.nlm.nih.gov/pubmed/?querykey=15&dbase=pubmed&querytype=eSearch&) | Search MAL-PDT | [85](http://www.ncbi.nlm.nih.gov/pubmed/?cmd=HistorySearch&querykey=15&) |
| [#14](http://www.ncbi.nlm.nih.gov/pubmed/?querykey=14&dbase=pubmed&querytype=eSearch&) | Search methyl aminolaevulinate | [180](http://www.ncbi.nlm.nih.gov/pubmed/?cmd=HistorySearch&querykey=14&) |
| [#13](http://www.ncbi.nlm.nih.gov/pubmed/?querykey=13&dbase=pubmed&querytype=eSearch&) | Search aminolevulinate | [7121](http://www.ncbi.nlm.nih.gov/pubmed/?cmd=HistorySearch&querykey=13&) |
| [#12](http://www.ncbi.nlm.nih.gov/pubmed/?querykey=12&dbase=pubmed&querytype=eSearch&) | Search metvix | [179](http://www.ncbi.nlm.nih.gov/pubmed/?cmd=HistorySearch&querykey=12&) |
| [#11](http://www.ncbi.nlm.nih.gov/pubmed/?querykey=11&dbase=pubmed&querytype=eSearch&) | Search kerastick | [16](http://www.ncbi.nlm.nih.gov/pubmed/?cmd=HistorySearch&querykey=11&) |
| [#10](http://www.ncbi.nlm.nih.gov/pubmed/?querykey=10&dbase=pubmed&querytype=eSearch&) | Search levulan | [5709](http://www.ncbi.nlm.nih.gov/pubmed/?cmd=HistorySearch&querykey=10&) |
| [#9](http://www.ncbi.nlm.nih.gov/pubmed/?querykey=9&dbase=pubmed&querytype=eSearch&) | Search ALA[tiab] | [28668](http://www.ncbi.nlm.nih.gov/pubmed/?cmd=HistorySearch&querykey=9&) |
| [#8](http://www.ncbi.nlm.nih.gov/pubmed/?querykey=8&dbase=pubmed&querytype=eSearch&) | Search protoporphyrins | [3900](http://www.ncbi.nlm.nih.gov/pubmed/?cmd=HistorySearch&querykey=8&) |
| [#7](http://www.ncbi.nlm.nih.gov/pubmed/?querykey=7&dbase=pubmed&querytype=eSearch&) | Search methyl 5-aminolevulinate | [166](http://www.ncbi.nlm.nih.gov/pubmed/?cmd=HistorySearch&querykey=7&) |
| [#6](http://www.ncbi.nlm.nih.gov/pubmed/?querykey=6&dbase=pubmed&querytype=eSearch&) | Search aminolevulinic acid | [5708](http://www.ncbi.nlm.nih.gov/pubmed/?cmd=HistorySearch&querykey=6&) |
| [#5](http://www.ncbi.nlm.nih.gov/pubmed/?querykey=5&dbase=pubmed&querytype=eSearch&) | Search photosensit*[tiab] | [14824](http://www.ncbi.nlm.nih.gov/pubmed/?cmd=HistorySearch&querykey=5&) |
| [#4](http://www.ncbi.nlm.nih.gov/pubmed/?querykey=4&dbase=pubmed&querytype=eSearch&) | Search PDT[tiab] | [5773](http://www.ncbi.nlm.nih.gov/pubmed/?cmd=HistorySearch&querykey=4&) |
| [#3](http://www.ncbi.nlm.nih.gov/pubmed/?querykey=3&dbase=pubmed&querytype=eSearch&) | Search photodynamic therapy | [13632](http://www.ncbi.nlm.nih.gov/pubmed/?cmd=HistorySearch&querykey=3&) |
| [#2](http://www.ncbi.nlm.nih.gov/pubmed/?querykey=2&dbase=pubmed&querytype=eSearch&) | Search photosensitizing agents | [27509](http://www.ncbi.nlm.nih.gov/pubmed/?cmd=HistorySearch&querykey=2&) |
| [#1](http://www.ncbi.nlm.nih.gov/pubmed/?querykey=1&dbase=pubmed&querytype=eSearch&) | Search photochemotherapy | [11313](http://www.ncbi.nlm.nih.gov/pubmed/?cmd=HistorySearch&querykey=1&) |

2. The Cochrane Library (issue 1, 2011)

| **Search** | | **Result** |
| --- | --- | --- |
| #1 | [(photochemotherapy):ti,ab,kw](http://onlinelibrary.wiley.com/o/cochrane/searchHistory?mode=runquery&qnum=1) | 572 |
| #2 | [(photosensitizing agents):ti,ab,kw](http://onlinelibrary.wiley.com/o/cochrane/searchHistory?mode=runquery&qnum=2) | 314 |
| #3 | [(photodynamic therapy):ti,ab,kw](http://onlinelibrary.wiley.com/o/cochrane/searchHistory?mode=runquery&qnum=3) | 614 |
| #4 | [(PDT):ti,ab,kw](http://onlinelibrary.wiley.com/o/cochrane/searchHistory?mode=runquery&qnum=4) | 413 |
| #5 | [(photosensit*):ti,ab,kw](http://onlinelibrary.wiley.com/o/cochrane/searchHistory?mode=runquery&qnum=5) | 612 |
| #6 | [(aminolevulinic acid):ti,ab,kw](http://onlinelibrary.wiley.com/o/cochrane/searchHistory?mode=runquery&qnum=6) | 212 |
| #7 | [(photosensit*):ti,ab,kw](http://onlinelibrary.wiley.com/o/cochrane/searchHistory?mode=runquery&qnum=7) | 612 |
| #8 | [(aminolevulinic acid):ti,ab,kw](http://onlinelibrary.wiley.com/o/cochrane/searchHistory?mode=runquery&qnum=8) | 212 |
| #9 | [(methyl 5-aminolevulinate):ti,ab,kw](http://onlinelibrary.wiley.com/o/cochrane/searchHistory?mode=runquery&qnum=9) | 5 |
| #10 | [(protoporphyrins):ti,ab,kw](http://onlinelibrary.wiley.com/o/cochrane/searchHistory?mode=runquery&qnum=10) | 67 |
| #11 | [(ALA):ti,ab,kw](http://onlinelibrary.wiley.com/o/cochrane/searchHistory?mode=runquery&qnum=11) | 375 |
| #12 | [(levulan):ti,ab,kw](http://onlinelibrary.wiley.com/o/cochrane/searchHistory?mode=runquery&qnum=12) | 3 |
| #13 | [(kerastick):ti,ab,kw](http://onlinelibrary.wiley.com/o/cochrane/searchHistory?mode=runquery&qnum=13) | 2 |
| #14 | [(metvix):ti,ab,kw](http://onlinelibrary.wiley.com/o/cochrane/searchHistory?mode=runquery&qnum=14) | 12 |
| #15 | [(aminolevulinate):ti,ab,kw](http://onlinelibrary.wiley.com/o/cochrane/searchHistory?mode=runquery&qnum=15) | 40 |
| #16 | [(methyl aminolaevulinate):ti,ab,kw](http://onlinelibrary.wiley.com/o/cochrane/searchHistory?mode=runquery&qnum=16) | 22 |
| #17 | [(mal-pdt):ti,ab,kw](http://onlinelibrary.wiley.com/o/cochrane/searchHistory?mode=runquery&qnum=17) | 38 |
| #18 | [(#1 OR #2 OR #3 OR #4 OR #5 OR #6 OR #7 OR #8 OR #9 OR #10 OR #11 OR #12 OR #13 OR #14 OR #15 OR #16 OR #17)](http://onlinelibrary.wiley.com/o/cochrane/searchHistory?mode=runquery&qnum=18) | 1578 |
| #19 | [(paget disease):ti,ab,kw](http://onlinelibrary.wiley.com/o/cochrane/searchHistory?mode=runquery&qnum=19) | 132 |
| #20 | [(paget's disease):ti,ab,kw](http://onlinelibrary.wiley.com/o/cochrane/searchHistory?mode=runquery&qnum=20) | 122 |
| #21 | [(paget):ti,ab,kw](http://onlinelibrary.wiley.com/o/cochrane/searchHistory?mode=runquery&qnum=21) | 134 |
| #22 | [(#18 AND #22)](http://onlinelibrary.wiley.com/o/cochrane/searchHistory?mode=runquery&qnum=22) | 0 |

3. Centre for Reviews and Dissemination (CRD) databases (DARE, HTA, NHS EED)

| **Search** | | **Result** |
| --- | --- | --- |
| 1 | [photochemotherapy OR photosensit* OR photodynamic OR PDT OR aminolevulinic AND acid OR aminolevulinate OR protoporphyrins OR ALA OR levulan OR kerastick OR metvix OR aminolaevulinate OR MAL-PDT](http://www.crd.york.ac.uk/CRDWeb/Search.aspx?SearchID=2936341&SessionID=2936341&D=62&E=53&H=72&SearchFor=%20photochemotherapy%20OR%20photosensit*%20OR%20photodynamic%20OR%20PDT%20OR%20aminolevulinic%20AND%20acid%20OR%20aminolevulinate%20OR%20protoporphyrins%20OR%20ALA%20OR%20levulan%20OR%20kerastick%20OR%20metvix%20OR%20aminolaevulinate%20OR%20MAL-PDT%20) | 187 |
| 2 | [paget's AND disease OR paget AND disease OR paget](http://www.crd.york.ac.uk/CRDWeb/Search.aspx?SearchID=2936343&SessionID=2936341&D=2&E=6&H=1&SearchFor=%20paget's%20AND%20disease%20OR%20paget%20AND%20disease%20OR%20paget%20) | 9 |
| 3 | [#1 AND #2](http://www.crd.york.ac.uk/CRDWeb/Search.aspx?SearchID=2936346&SessionID=2936341&D=0&E=0&H=0&SearchFor=#1 AND #2) | 0 |

4. EMBASE (Ovid) 1980 – 2011 (week 4)

| **Search** | | **Result** |
| --- | --- | --- |
| 1 | exp photochemotherapy/ | 4612 |
| 2 | exp photosensitizing agent/ | 24972 |
| 3 | exp photodynamic therapy/ | 11099 |
| 4 | PDT.ti,ab. | 6620 |
| 5 | exp aminolevulinic acid/ | 4880 |
| 6 | exp aminolevulinic acid methyl ester/ | 592 |
| 7 | exp protoporphyrin/ | 3788 |
| 8 | ALA.ti,ab. | 29061 |
| 9 | levulan.mp. | 254 |
| 10 | kerastick.mp. | 89 |
| 11 | metvix.mp. | 350 |
| 12 | aminolevulinate.ti,ab. | 1122 |
| 13 | methyl aminolaevulinate.ti,ab. | 38 |
| 14 | MAL-PDT.ti,ab. | 92 |
| 15 | 1 or 2 or 3 or 4 or 5 or 6 or 7 or 8 or 9 or 10 or 11 or 12 or 13 or 14 | 64908 |
| 16 | exp paget skin disease/ | 1536 |
| 17 | paget disease.mp. | 575 |
| 18 | exp paget nipple disease/ | 5350 |
| 19 | 16 or 17 or 18 | 7165 |
| 20 | 15 and 19 | 89 |

|  |
| --- |

5. CINAHL (EbscoHost)

| **Search** | | **Result** |
| --- | --- | --- |
| S17 | S12 and S16 | 1 |
| S16 | S13 or S14 or S15 | 362 |
| S15 | paget | 105 |
| S14 | paget's disease | 262 |
| S13 | paget disease | 50 |
| S12 | S1 or S2 or S3 or S4 or S5 or S6 or S7 or S8 or S9 or S10 or S11 | 1055 |
| S11 | MAL-PDT | 3 |
| S10 | aminolevulinate | 22 |
| S9 | metvix | 0 |
| S8 | kerastick | 0 |
| S7 | levulan | 0 |
| S6 | protoporphyrins | 0 |
| S5 | methyl 5-aminolevulinate | 11 |
| S4 | aminolevulinic acid | 71 |
| S3 | TI PDT or AB PDT | 208 |
| S2 | photodynamic therapy | 719 |
| S1 | MH photochemotherapy or MH photosensitizing agents | 474 |

6. Web of Science (ISI Web of Knowledge)

| **Search** | **Result** |
| --- | --- |
| Topic=((photodynamic OR photosensit* OR levulan OR kerastick OR metvix OR ALA OR PDT OR MAL-PDT OR aminolevulinate OR aminolaevulinate OR protoporphyrin*)) AND Topic=("paget's disease" OR "paget disease" OR paget*)  Timespan=All Years. Databases=SCI-EXPANDED, SSCI, A&HCI, CPCI-S, CPCI-SSH. | 46 |

7. PsycINFO (Ovid) (1806 to January, Week 4, 2011)

| **Search** | | **Result** |
| --- | --- | --- |
| 1 | photosensitizing.mp. | 4 |
| 2 | photodynamic therapy.mp. | 8 |
| 3 | PDT.ti,ab. | 95 |
| 4 | ALA.ti,ab. | 539 |
| 5 | aminolevulinic acid.mp. | 19 |
| 6 | aminolevulinate.ti,ab. | 6 |
| 7 | aminolaevulinate.ti,ab. | 0 |
| 8 | 1 or 2 or 3 or 4 or 5 or 6 or 7 | 657 |
| 9 | paget's disease.mp. | 22 |
| 10 | paget disease.mp. | 8 |
| 11 | paget.mp. | 44 |
| 12 | 9 or 10 or 11 | 66 |
| 13 | 8 and 12 | 0 |

### Grey literature searches

Search terms: Paget OR Paget’s (unless otherwise noted)

- National Guideline Clearinghouse

- CMA Infobase

- ClinicalTrials.gov

- Google.ca (paget OR paget's) AND (PDT OR photodynamic) – scanned first 200 hits only

- VulvaPagetsSupport.org (RefWorks bibliography)

- relevant web sites on the Canadian Agency for Drugs and Technologies in Health (CADTH) checklist

Conference proceedings

- American Society for Laser Medicine and Surgery (2004-2010)

- British Association of Dermatologists (2004-2010)

- Canadian Dermatology Association (2005-2010)

- American Academy of Dermatology (2004-2010)

- European Academy of Dermatology and Venereology (EADV) (2004-2005; not available from 2006 on)

- European Society for Photodynamic Therapy (2005, [2006 not available], 2007, 2008, 2009, 2010)
