# Supplementary material for: Effectiveness of photodynamic therapy for mammary and extra-mammary Paget's disease: a state of the science review
Source: BMC Dermatol. 2011 Jun 15;11:13. doi: 10.1186/1471-5945-11-13 (PMC3141658; doi:10.1186/1471-5945-11-13)
Supplement: Additional file 2 — Table of included studies of photodynamic therapy (PDT) for Paget's disease. Details of study and patient characteristics, interventions, outcomes and quality of included studies are provided in additional file 2. [file 1471-5945-11-13-S2.DOCX]

### Additional file 2 - Table of included studies of photodynamic therapy (PDT) for Paget’s disease

| **Study Authors (year published)** | **Study Design** | **Patients** | **Intervention** | **Outcome Measures** | **Findings** | **Oxford Level of Evidence** |
| --- | --- | --- | --- | --- | --- | --- |
| **Prospective case series** | | | | | | |
| - **Li et al. (2010)**      - China - EMPD | *Design*:  prospective case series  *Setting*:  single-centre, academic clinic  *Number of patients*: 16 | *Patient characteristics*  *Gender*: 14 male, 2 female  *Age*:  mean (range): 68.2 (54-83) years  *Length of Follow-up*: 24 months  *Lesion characteristics*  *No. of lesions*: 21  *Location of lesion(s):*  ∙9/21 scrotum  ∙5/21 axillary  ∙5/21 perianal  ∙2/21 vulval  *Size of lesion(s)*:  ∙mean (range): 3.7 (1-12) cm  *History*  *Metastases*: None; however 1 perianal PD lesion was associated with underlying colorectal cancer  *Prior treatment and response to treatment*:   - NR | PDT:  *Skin Prep*: crusts removed with curette  *Drug*: 20% 5-ALA  *Dosage*: applied on lesion + 20mm clinically disease-free margin  *Route of Admin*.: topical  *Time to Photoact.*: 6 hours  *Light Source*: red light, Led lamp (Omnilux), 633nm  *Light Dose*: 113 J/cm^2^ per tx. (339  *Light Intensity*: 126 mW/cm^2^  *Treatment Time*: 15 minutes (2*7.5 min separated by 30 min)  *No. of Treatments*: 3 (1 week apart) | *Outcomes*:  Lesion response  -clinical and/or histological exam; CR = disappearance of lesion | *Outcomes*:  -after last tx.: clinical CR 14/21 lesions  -at 6 months: CR 11/21 (53%)  scrotal-7/9 (78%), axillary-2/5 (40%), perianal-2/5 (40%), vulval-0/2 (0%)  <4cm-6/8 (75%), 4-8cm-4/6 (67%), >8cm-1/7 (14%)  -at 12 months: CR 9/21 (43%)  scrotal-6/9 (67%), axillary-2/5 (40%), perianal-1/5 (20%), vulval-0/2 (0%)  <4cm-6/8 (75%), 4-8cm-3/6 (50%), >8cm-0/7 (0%)  -at 24 months: CR 7/21 (33%)  scrotal-6/9 (67%), axillary-1/5 (20%), perianal-0/5 (0%), vulval-0/2 (0%)  <4cm-5/8 (63%), 4-8cm-2/6 (33%), >8cm-0/7 (0%)  - over 24 month period, 7/21 (33%) had PR (2 axillary, 4 perianal, 1 vulval) and 7/14 CR lesions recurred (2 scrotal, 2 axillary, 1 perianal, 1 vulval); these PR and recurrent lesions (14) were treated with alternate therapies | 4 |
|  |  |  |  | Cosmetic outcome  -investigator- (by blinded observer) and patient-assessed; graded as excellent (no hypertrophic scarring, atrophy or induration, no redness, no pigmentation), good (no hypertrophic scarring, atrophy or induration but slight redness or pigmentation), fair (slight to moderate hypertrophic scarring, atrophy or induration, moderate redness or pigmentation), or poor (extensive hypertrophic scarring, atrophy and induration or serious redness or obvioius pigmentation) | investigator-assessed:  - 5/14 (36%) lesions with incomplete response were rated as good or excellent  - 9/14 (64%) rated as poor for atrophy (2), induration (1), depigmentation (4), redness (2)  patient-assessed:  - 7 patients (100%) with complete response satisfied with cosmetic outcome |  |
|  |  |  |  | *Adverse Events*:  -pain assessed using VAS scale (0-none, 10-extreme)  -local and systemic phototoxic reactions up to 3 months reported and graded via NCICTC  -an other AE documented | *Adverse Events*:  - pain: mean VAS ± SD (range): 5.4 ± 1.3 (2-10); perianal 9.1, vulval 6.5, axillary 4.5, scrotal 3.2  - 5 patients could not endure and received anesthesia  - all patients reported specific skin reactions to PDT during/after illumination, mild-moderate erythema and edema and eriosion and crusting 24 hours after treatment (disappeared within 2 weeks)  - 2/3’s of patients reported slight to moderate discomfort during illumination (itching, burning…)  - no serious AEs or death |  |
| *Notes:*  -16 patients (21 lesions) with histologically verified EMPD recruited from dermatology clinic – all Asian with Fitzpatrick skin type IV/V  - patients with metastisis or invasive EMPD, with tumour thickness >2mm, with poor general health or poor compliance, who are pregnant or breastfeeding or have hade other treatments within the past 6 months were excluded  - lesions with noncomplete responses at 6 months after last treatment received surgical excision, cryotherapy or CO_2_ laser therapy  - overall longterm CR rate (24 months) 33%; recurrence rate 50%  - CR rate was correlated with tumour location and size (but not thickness): scrotal lesions showed better response to PDT at 6, 12 and 24 months compared to axillary, perianal and vulval lesions | | | | | | |
| - **Raspagliesi et al. (2006)** - Italy - EMPD | *Design*:  prospective  case series  *Setting*:  single-centre,  academic clinic  *Number of patients*: 7 | *Patient characteristics*  *Patient no.*: 1  *Gender*: female  *Age*: 55 years  *Length of Follow-up*: 5 months  *Lesion characteristics*  *No. of lesions*: 2  *Location of lesion(s):*  ∙mucosal vulva  ∙perianal  *Size of lesion(s)*:  ∙5cm x 4cm (20cm^2^)  ∙3.5cm x 3cm (10.5cm^2^)  *History*  *Metastases*: NR  *Prior treatment and response to treatment*:   - 2 surgical excisions, 4 laser excisions 🡪 recurrence x 2 | PDT:  *Skin Prep*: saline rinse  *Drug*: 16% m-ALA (Metvix®)  *Dosage*: 1 g (mean)  *Route of Admin*.: topical  *Time to Photoact.*: 3 hours  *Light Source*: red light, Led lamp (Aktlite 128), 630nm  *Light Dose*: NR  *Light Intensity*: 37 J/cm^2^  *Treatment Time*: 10 minutes  *No. of Treatments*: 3 (every 3 weeks) | *Outcomes*:  Lesion response  -clinical and histological (biopsy) exam | *Outcomes*:  -vulva: CR (histological confirmation not available)  -perianal: NC (no change) | 4 |
|  |  |  |  | Cosmetic outcome  -baseline function, anatomic profile, scarring | -“acceptable”, no substantial changes in function or profile, no post-treatment scarring observed |  |
|  |  |  |  | *Adverse Events*:  -treatment-related pain, infection control, local pruritus, observed or reported phototoxicity | *Adverse Events*:  -pain: pre-medicated with NSAIDs (nimesulida 100mg); patient reported discomfort  -infection control: topical antibiotic applied to treatment area immediately after treatment and during the first 3-4 days post-treatment; no infections reported  -local pruritus minimal after last MAL-PDT application  -“expected local phototoxicity” reported |  |
|  |  | *Patient characteristics*  *Patient no.*: 2  *Gender*: female  *Age*: 60 years  *Length of Follow-up*: 4 months  *Lesion characteristics*  *No. of lesions*: 1  *Location of lesion(s):*  mucosal vulva  *Size of lesion(s)*:  2cm x 7cm (14cm^2^)  *History*  *Metastases*: NR  *Prior treatment and response to treatment*:   - 8 laser excisions 🡪 recurrence x 8 | PDT:  *Skin Prep*: saline rinse  *Drug*: 16% m-ALA (Metvix®)  *Dosage*: 1 g (mean)  *Route of Admin*.: topical  *Time to Photoact.*: 3 hours  *Light Source*: red light, Led lamp (Aktlite 128), 630nm  *Light Dose*: NR  *Light Intensity*: 37 J/cm^2^  *Treatment Time*: 10 minutes  *No. of Treatments*: 3 (every 3 weeks) | *Outcomes*:  Lesion response  - clinical and histological (biopsy) exam | *Outcomes*:  -vulva: NC (no change) |  |
|  |  |  |  | Cosmetic outcome  -baseline function, anatomic profile, scarring | -“acceptable”, no substantial changes in function or profile, no post-treatment scarring observed |  |
|  |  |  |  | *Adverse Events*:  -treatment-related pain, infection control, local pruritus, observed or reported phototoxicity | *Adverse Events*:  -pain: pre-medicated with NSAIDs (nimesulida 100mg); patient reported discomfort; persistent pain for 5-6 days post-treatment (required analgesic x 5 days)  -infection control: topical antibiotic applied to treatment area immediately after treatment and during the first 3-4 days post-treatment; no infections reported  -local pruritus minimal after last MAL-PDT application  -“expected local phototoxicity” reported |  |
|  |  | *Patient characteristics*  *Patient no.*: 3  *Gender*: female  *Age*: 57 years  *Length of Follow-up*: 4 months  *Lesion characteristics*  *No. of lesions*: 2  *Location of lesion(s):*  ∙mucosal vulva  ∙perianal  *Size of lesion(s)*:  ∙3.5cm x 2cm (7cm^2)^  ∙1.5cm x 1cm (1.5cm^2)^  *History*  *Metastases*: NR  *Prior treatment and response to treatment*:   - 1 surgical excision, 3 laser excisions 🡪 recurrence x 1 | PDT:  *Skin Prep*: saline rinse  *Drug*: 16% m-ALA (Metvix®)  *Dosage*: 1 g (mean)  *Route of Admin*.: topical  *Time to Photoact.*: 3 hours  *Light Source*: red light, Led lamp (Aktlite 128), 630nm  *Light Dose*: NR  *Light Intensity*: 37 J/cm^2^  *Treatment Time*: 10 minutes  *No. of Treatments*: 3 (every 3 weeks) | *Outcomes*:  Lesion response  - clinical and histological (biopsy) exam | *Outcomes*:  -vulva: CR  -perianal: CR |  |
|  |  |  |  | Cosmetic outcome  -baseline function, anatomic profile, scarring | -“acceptable”, no substantial changes in function or profile, no post-treatment scarring observed |  |
|  |  |  |  | *Adverse Events*:  -treatment-related pain, infection control, local pruritus, observed or reported phototoxicity | *Adverse Events*:  -pain: pre-medicated with NSAIDs (nimesulida 100mg); patient reported discomfort  -infection control: topical antibiotic applied to treatment area immediately after treatment and during the first 3-4 days post-treatment; no infections reported  -local pruritus minimal after last MAL-PDT application  -“expected local phototoxicity” reported |  |
|  |  | *Patient characteristics*  *Patient no.*: 4  *Gender*: female  *Age*: 67 years  *Length of Follow-up*: 4 months  *Lesion characteristics*  *No. of lesions*: 2  *Location of lesion(s):*  ∙mucosal vulva  ∙perianal  *Size of lesion(s)*:  ∙6cm x 3cm (18cm^2^)  ∙4cm x 3cm (12cm^2^)  *History*  *Metastases*: NR  *Prior treatment and response to treatment*:   - 2 surgical excisions 🡪 recurrence x 2 | PDT:  *Skin Prep*: saline rinse  *Drug*: 16% m-ALA (Metvix®)  *Dosage*: 1 g (mean)  *Route of Admin*.: topical  *Time to Photoact.*: 3 hours  *Light Source*: red light, Led lamp (Aktlite 128), 630nm  *Light Dose*: NR  *Light Intensity*: 37 J/cm^2^  *Treatment Time*: 10 minutes  *No. of Treatments*: 3 (every 3 weeks) | *Outcomes*:  Lesion response  - clinical and histological (biopsy) exam | *Outcomes*:  -vulva: CR  -perianal: CR |  |
|  |  |  |  | Cosmetic outcome  -baseline function, anatomic profile, scarring | -“acceptable”, no substantial changes in function or profile, no post-treatment scarring observed |  |
|  |  |  |  | *Adverse Events*:  -treatment-related pain, infection control, local pruritus, observed or reported phototoxicity | *Adverse Events*:  -pain: pre-medicated with NSAIDs (nimesulida 100mg); patient reported discomfort; persistent pain for 5-6 days post-treatment (required analgesic x 5 days)  -infection control: topical antibiotic applied to treatment area immediately after treatment and during the first 3-4 days post-treatment; no infections reported  -local pruritus minimal after last MAL-PDT application  -“expected local phototoxicity” reported |  |
|  |  | *Patient characteristics*  *Patient no.*: 5  *Gender*: female  *Age*: 59 years  *Length of Follow-up*: 2 months  *Lesion characteristics*  *No. of lesions*:1  *Location of lesion(s):*  mucosal vulva  *Size of lesion(s)*:  5cm x 3cm (15cm^2^)  *History*  *Metastases*: NR  *Prior treatment and response to treatment*:   - 2 surgical excisions, 4 laser excisions 🡪 recurrence x 5 | PDT:  *Skin Prep*: saline rinse  *Drug*: 16% m-ALA (Metvix®)  *Dosage*: 1 g (mean)  *Route of Admin*.: topical  *Time to Photoact.*: 3 hours  *Light Source*: red light, Led lamp (Aktlite 128), 630nm  *Light Dose*: NR  *Light Intensity*: 37 J/cm^2^  *Treatment Time*: 10 minutes  *No. of Treatments*: 3 (every 3 weeks) | *Outcomes*:  Lesion response  -clinical and histological (biopsy) exam | *Outcomes*:  -vulva: CR |  |
|  |  |  |  | Cosmetic outcome  -baseline function, anatomic profile, scarring | -“acceptable”, no substantial changes in function or profile, no post-treatment scarring observed |  |
|  |  |  |  | *Adverse Events*:  -treatment-related pain, infection control, local pruritus, observed or reported phototoxicity | *Adverse Events*:  -pain: pre-medicated with NSAIDs (nimesulida 100mg); patient reported discomfort  -infection control: topical antibiotic applied to treatment area immediately after treatment and during the first 3-4 days post-treatment; no infections reported  -local pruritus minimal after last MAL-PDT application  -“expected local phototoxicity” reported |  |
|  |  | *Patient characteristics*  *Patient no.*: 6  *Gender*: female  *Age*: 69 years  *Length of Follow-up*: 1 months  *Lesion characteristics*  *No. of lesions*: 1  *Location of lesion(s):*  mucosal vulva  *Size of lesion(s)*:  2.5cm x 2.5cm (6.25cm^2^)  *History*  *Metastases*: NR  *Prior treatment and response to treatment*:   - 1 surgical excision, 2 laser excisions 🡪 recurrence x 2 | PDT:  *Skin Prep*: saline rinse  *Drug*: 16% m-ALA (Metvix®)  *Dosage*: 1 g (mean)  *Route of Admin*.: topical  *Time to Photoact.*: 3 hours  *Light Source*: red light, Led lamp (Aktlite 128), 630nm  *Light Dose*: NR  *Light Intensity*: 37 J/cm^2^  *Treatment Time*: 10 minutes  *No. of Treatments*: 3 (every 3 weeks) | *Outcomes*:  Lesion response  -clinical and histological (biopsy) exam | *Outcomes*:  -vulva: CR |  |
|  |  |  |  | Cosmetic outcome  -baseline function, anatomic profile, scarring | -“acceptable”, no substantial changes in function or profile, no post-treatment scarring observed |  |
|  |  |  |  | *Adverse Events*:  -treatment-related pain, infection control, local pruritus, observed or reported phototoxicity | *Adverse Events*:  -pain: pre-medicated with NSAIDs (nimesulida 100mg); patient reported discomfort  -infection control: topical antibiotic applied to treatment area immediately after treatment and during the first 3-4 days post-treatment; no infections reported  -local pruritus minimal after last MAL-PDT application  -“expected local phototoxicity” reported |  |
|  |  | *Patient characteristics*  *Patient no.*: 7  *Gender*: female  *Age*: 75 years  *Length of Follow-up*: 1 months  *Lesion characteristics*  *No. of lesions*: 2  *Location of lesion(s):*  ∙cutaneous vulva  ∙axilla  *Size of lesion(s)*:  ∙6cm x 3cm (18cm^2^)  ∙4.5cm x 4cm (18cm^2^)  *History*  *Metastases*: NR  *Prior treatment and response to treatment*:   - 1 laser excision 🡪 recurrence x 2 | PDT:  *Skin Prep*: saline rinse  *Drug*: 16% m-ALA (Metvix®)  *Dosage*: 1 g (mean)  *Route of Admin*.: topical  *Time to Photoact.*: 3 hours  *Light Source*: red light, Led lamp (Aktlite 128), 630nm  *Light Dose*: NR  *Light Intensity*: 37 J/cm^2^  *Treatment Time*: 10 minutes  *No. of Treatments*: 3 (every 3 weeks) | *Outcomes*:  Lesion response  -clinical and histological (biopsy) exam | *Outcomes :*  -vulva: PR  -axilla: CR |  |
|  |  |  |  | Cosmetic outcome  -baseline function, anatomic profile, scarring | -“acceptable”, no substantial changes in function or profile, no post-treatment scarring observed |  |
|  |  |  |  | *Adverse Events*:  -treatment-related pain, infection control, local pruritus, observed or reported phototoxicity | *Adverse Events*:  -pain: pre-medicated with NSAIDs (nimesulida 100mg); patient reported discomfort  -infection control: topical antibiotic applied to treatment area immediately after treatment and during the first 3-4 days post-treatment; no infections reported  -local pruritus minimal after last MAL-PDT application  -“expected local phototoxicity” reported |  |
| *Notes:*  -7 female patients (all Caucasian) with recurrent Paget’s of the vulva and region after conventional treatments (vulvectomy or wide vulvar resection or laser ablation)  -**summary**: 7 female patients with EMPD (11 lesions total), mean age ± SD: 63 ± 7.3 years (range: 50-75), treated with MAL-PDT: 3 treatments (3 weeks apart) for local recurrence after conventional treatment failure; results: after 3 treatments: mucosal vulva-CR 5/6 (83%), NC 1/6 (17%); cutaneous vulva-PR 1/1 (100%); perianal-CR 1/3 (33%), PR 1/3 (33%), NC 1/3 (33%); axilla-CR 1/1; overall-CR 8/11 (73%); PR 1/11 (9%); NC 2/11 (18%); follow-up from 1-5 months after treatment | | | | | | |
| **Retrospective case reports/case series** | | | | | | |
| - **Housel et al. (2010)** and **Shieh et al. (2002)** - USA - EMPD | *Design*:  retrospective  case series  *Setting*:  single-centre,  academic clinic  *Number of patients*: 5 | *Patient characteristics*  *Patient no.*: 1  *Gender*: male  *Age*: 75 years  *Length of Follow-up*: 19 months  *Lesion characteristics*  *No. of lesions*: 1  *Location of lesion(s):* axilla  *Size of lesion(s)*:  8.5cm x 3.3cm (28.05cm^2^)  *History*  *Metastases*: None  *Prior treatment and response to treatment*:   - excision 🡪 recurrence | PDT:  *Skin Prep*: 1% lignocaine  *Drug*: 20% 5-ALA  *Dosage*: 20-30mg/cm^2^ applied on lesion + 1.0cm clinically disease-free margin  *Route of Admin*.: topical  *Time to Photoact.*: NR  *Light Source*: argon dye laser, 632.8nm (occasionally red lamp, 590-700nm used prior to laser)  *Light Dose*: 200-300 J/cm^2^ (red lamp-100-200 J/cm^2^)  *Light Intensity*: 80-150 mW/cm^2^ (red lamp-42-80 mW/cm^2^)  *Treatment Time*: NR  *No. of Treatments*: 1 | *Outcomes*:  Lesion response  - clinical and histological (2 biopsies) exam  -CR = 100% disease clearance, PR = 50-99% clearance, MR = < 50% clearance (minimal response)  -recurrence: clinical assessment after CR | *Outcomes*: (per lesion)  axilla  -at 6 months: PR (no re-treatment)  -recurrence: N/A | 4 |
|  |  |  |  | Cosmetic Outcome  -clinical assessment | -no scarring  -"excellent" cosmetic and anatomical function |  |
|  |  |  |  | *Adverse Events*:  -erythema and swelling; clinical examination | *Adverse Events*:  -transient in nature, no treatment required  -healing in 2-3 weeks post treatment |  |
|  |  | *Patient characteristics*  *Patient no.*: 2  *Gender*: male  *Age*: 50 years  *Length of Follow-up*: 99 months  *Lesion characteristics*  *No. of lesions*: 1  *Location of lesion(s):* L groin  *Size of lesion(s)*:  5.5cm x 2.5cm (13.75cm^2^)  *History*  *Metastases*: None  *Prior treatment and response to treatment*:   - none | PDT:  Treatment 1  *Skin Prep*: 1% lignocaine  *Drug*: 20% 5-ALA  *Dosage*: 20-30mg/cm^2^ applied on lesion + 1.0cm clinically disease-free margin  *Route of Admin*.: topical  *Time to Photoact.*: NR  *Light Source*: argon dye laser, 632.8nm (occasionally red lamp, 590-700nm used prior to laser)  *Light Dose*: 200-300 J/cm^2^ (red lamp-100-200 J/cm^2^)  *Light Intensity*: 80-150 mW/cm^2^ (red lamp-42-80 mW/cm^2^)  *Treatment Time*: NR  *No. of Treatments*: 5  Treatment 2  *Skin Prep*: 1% lignocaine  *Drug*: Porfimer sodium (Photofrin)  *Dosage*: 1mg/kg  *Route of Admin*.: intravenous  *Time to Photoact.*: 48 hours  *Light Source*: argon dye laser, 632.8nm  *Light Dose*: 215 J/cm^2^  *Light Intensity*: 150 mW/cm^2^  *Treatment Time*: NR  *No. of Treatments*: 1 | *Outcomes*:  Lesion response  - clinical and histological (2 biopsies) exam  -CR = 100% disease clearance, PR = 50-99% clearance, MR = < 50% clearance (minimal response)  -recurrence: clinical assessment after CR | *Outcomes*: (per lesion)  L groin  -at 6 months: PR; after repeat ALA-PDT (4): PR  -recurrence: N/A  -after 1 Porfimer sodium PDT: CR (no re-treatment)  -recurrence: none at 12 FU after Porfimer sodium PDT |  |
|  |  |  |  | Cosmetic Outcome  -clinical assessment | -no scarring  -"excellent" cosmetic and anatomical function |  |
|  |  |  |  | *Adverse Events*:  -erythema and swelling; clinical examination | *Adverse Events*:  -transient in nature, no treatment required  -healing in 2-3 weeks post treatment |  |
|  |  | *Patient characteristics*  *Patient no.*: 3  *Gender*: male  *Age*: 61 years  *Length of Follow-up*: 20 months  *Lesion characteristics*  *No. of lesions*: 3  *Location of lesion(s):*  ∙R pubis  ∙penile base and shaft  ∙R scrotum  *Size of lesion(s)*:  ∙6.5cm x 7.0cm (45.5cm^2^)  ∙7.0cm x 7.0cm (49.0cm^2^)  ∙7.5cm x 6.0cm (45.0cm^2^)  *History*  *Metastases*: None  *Prior treatment and response to treatment*:   - laser excision (all) 🡪 recurrence | PDT:  *Skin Prep*: 1% lignocaine  *Drug*: 20% 5-ALA  *Dosage*: 20-30mg/cm^2^ applied on lesion + 1.0cm clinically disease-free margin  *Route of Admin*.: topical  *Time to Photoact.*: NR  *Light Source*: argon dye laser, 632.8nm (occasionally red lamp, 590-700nm used prior to laser)  *Light Dose*: 200-300 J/cm^2^ (red lamp-100-200 J/cm^2^)  *Light Intensity*: 80-150 mW/cm^2^ (red lamp-42-80 mW/cm^2^)  *Treatment Time*: NR  *No. of Treatments*: 3 | *Outcomes*:  Lesion response  - clinical and histological (2 biopsies) exam  -CR = 100% disease clearance, PR = 50-99% clearance, MR = < 50% clearance (minimal response)  -recurrence: clinical assessment after CR | *Outcomes*: (per lesion)  R pubis  -at 6 months: PR, after repeat ALA-PDT (2): PR  -recurrence: N/A  penile base/shaft  -at 6 months: MR (minimal response), after repeat ALA-PDT (2): PR  -recurrence: N/A  R scrotum  -at 6 months: MR, after repeat ALA-PDT (2): PR  -recurrence: N/A |  |
|  |  |  |  | Cosmetic Outcome  -clinical assessment | -no scarring  -"excellent" cosmetic and anatomical function |  |
|  |  |  |  | *Adverse Events*:  -erythema and swelling; clinical examination | *Adverse Events*:  -transient in nature, no treatment required  -healing in 2-3 weeks post treatment |  |
|  |  | *Patient characteristics*  *Patient no.*: 4  *Gender*: male  *Age*: 65 years  *Length of Follow-up*: 6-9 months  *Lesion characteristics*  *No. of lesions*: 6  *Location of lesion(s):*  ∙L pubis  ∙L buttock  ∙R pubis  ∙R scrotum-superior  ∙R scrotum-middle  ∙R scrotum-inferior  *Size of lesion(s)*:  ∙<1.5cm  ∙<2.0cm  ∙<1.5cm  ∙<1.5cm  ∙<1.5cm  ∙<1.5cm  *History*  *Metastases*: None  *Prior treatment and response to treatment*:   - laser excision (all but R inf scrotum) 🡪 recurrence - R inf scrotum – no prior treatment | PDT:  *Skin Prep*: 1% lignocaine  *Drug*: 20% 5-ALA  *Dosage*: 20-30mg/cm^2^ applied on lesion + 1.0cm clinically disease-free margin  *Route of Admin*.: topical  *Time to Photoact.*: NR  *Light Source*: argon dye laser, 632.8nm (occasionally red lamp, 590-700nm used prior to laser)  *Light Dose*: 200-300 J/cm^2^ (red lamp-100-200 J/cm^2^)  *Light Intensity*: 80-150 mW/cm^2^ (red lamp-42-80 mW/cm^2^)  *Treatment Time*: NR  *No. of Treatments*: 1-2 | *Outcomes*:  Lesion response  - clinical and histological (2 biopsies) exam  -CR = 100% disease clearance, PR = 50-99% clearance, MR = < 50% clearance (minimal response)  -recurrence: clinical assessment after CR | *Outcomes*: (per lesion)  L pubis  -at 6 months: CR (no retreatment)  -recurrence: none  L buttock  -at 6 months: CR (no retreatment)  -recurrence: none  R pubis  -at 6 months: CR (no retreatment)  -recurrence: none  R scrotum (sup)  -at 6 months: CR; after repeat ALA-PDT (1): CR  -recurrence: at 9 months (after re-treatment CR)  R scrotum (mid)  -at 6 months: MR; after repeat ALA-PDT (1): CR  -recurrence: N/A  R scrotum (inf)  -at 6 months: MR; after repeat ALA-PDT (1): CR  -recurrence: N/A |  |
|  |  |  |  | Cosmetic Outcome  -clinical assessment | -no scarring  -"excellent" cosmetic and anatomical function |  |
|  |  |  |  | *Adverse Events*:  -erythema and swelling; clinical examination | *Adverse Events*:  -transient in nature, no treatment required  -healing in 2-3 weeks post treatment |  |
|  |  | *Patient characteristics*  *Patient no.*: 5  *Gender*: male  *Age*: 72 years  *Length of Follow-up*: 4-88 months  *Lesion characteristics*  *No. of lesions*: 5  *Location of lesion(s):*  ∙distal penis  ∙midline penis  ∙proximal penis  ∙L scrotum (sup)  ∙L scrotum (inf)  *Size of lesion(s)*:  ∙2.0cm x 2.0cm (4.0cm^2^)  ∙2.0cm x 1.2cm (2.4cm^2^)  ∙5.5cm x 5.0cm (27.5cm^2^)  ∙1.5cm x 1.3cm (1.95cm^2^)  ∙1.3cm x 0.7cm (0.91cm^2^)  *History*  *Metastases*: None  *Prior treatment and response to treatment*:   - none in penis - MMS in scrotum | PDT:  *Skin Prep*: 1% lignocaine  *Drug*: 20% 5-ALA  *Dosage*: 20-30mg/cm^2^ applied on lesion + 1.0cm clinically disease-free margin  *Route of Admin*.: topical  *Time to Photoact.*: NR  *Light Source*: argon dye laser, 632.8nm (occasionally red lamp, 590-700nm used prior to laser)  *Light Dose*: 200-300 J/cm^2^ (red lamp-100-200 J/cm^2^)  *Light Intensity*: 80-150 mW/cm^2^ (red lamp-42-80 mW/cm^2^)  *Treatment Time*: NR  *No. of Treatments*: 1-3 | *Outcomes*:  Lesion response  - clinical and histological (2 biopsies) exam  -CR = 100% disease clearance, PR = 50-99% clearance, MR = < 50% clearance (minimal response)  -recurrence: clinical assessment after CR | *Outcomes*: (per lesion)  distal penis  -at 6 months: CR (no retreatment)  -recurrence: none  mid penis  -at 6 months: MR; after repeat ALA-PDT (2): PR  -recurrence: N/A  prox penis  -at 6 months: CR (no retreatment)  -recurrence: none  L scrotum (sup)  -at 6 months: CR; after repeat ALA-PDT (1): REC  -recurrence: at 10 months (after re-treatment recurred again at 6 months)  L scrotum (inf)  -at 6 months: CR; after repeat ALA-PDT (1): REC  -recurrence: at 10 months (after re-treatment recurred again at 6 months) |  |
|  |  |  |  | Cosmetic Outcome  -clinical assessment | -no scarring  -"excellent" cosmetic and anatomical function |  |
|  |  |  |  | *Adverse Events*:  -erythema and swelling; clinical examination | *Adverse Events*:  -transient in nature, no treatment required  -healing in 2-3 weeks post treatment |  |
|  |  | *Patient characteristics*  *Patient no.*: 6  *Gender*: female  *Age*: 78 years  *Length of Follow-up*: 48-68 months  *Lesion characteristics*  *No. of lesions*: 3  *Location of lesion(s):*  ∙R perianal  ∙L perianal  ∙posterior perianal  *Size of lesion(s)*:  ∙6.5cm x 6.5cm (42.25cm^2^)  ∙6.0cm x 6.0cm (36.0cm^2^)  ∙1.0cm x 1.0cm (1.0cm^2^)  *History*  *Metastases*: None  *Prior treatment and response to treatment*:   - excision and ALA-PDT on R perianal 🡪 recurrence - ALA-PDT on L and posterior perianal 🡪 recurrence | PDT:  *Skin Prep*: 1% lignocaine  *Drug*: Porfimer sodium (Photofrin)  *Dosage*: 1mg/kg  *Route of Admin*.: intravenous  *Time to Photoact.*: 48 hours  *Light Source*: argon dye laser, 632.8nm  *Light Dose*: 215 J/cm^2^  *Light Intensity*: 150 mW/cm^2^  *Treatment Time*: NR  *No. of Treatments*: 1  *note: performed in OR under general anesthesia | *Outcomes*:  Lesion response  - clinical and histological (2 biopsies) exam  -CR = 100% disease clearance, PR = 50-99% clearance, MR = < 50% clearance (minimal response)  -recurrence: clinical assessment after CR | *Outcomes*: (per lesion)  R perianal  -at 6 months: CR (no retreatment)  -recurrence: none  L perianal  -at 6 months: PR (no retreatment)  -recurrence: N/A  Posterior perianal  -at 6 months: PR (no retreatment)  -recurrence: N/A |  |
|  |  |  |  | Cosmetic Outcome  -clinical assessment | -greater incidence of mild scarring in pts. treated with Porfimer-sodium-PDT, but still less severe than prior surgical scars  -no functional impairments |  |
|  |  |  |  | *Adverse Events*:  -erythema and swelling; clinical examination | *Adverse Events*:  -no major adverse events  -healing in 2-3 weeks post treatment (back to work) and complete healing in 3 months |  |
|  |  | *Patient characteristics*  *Patient no.*: 7  *Gender*: female  *Age*: 80 years  *Length of Follow-up*: 12 months  *Lesion characteristics*  *No. of lesions*: 3  *Location of lesion(s):*  ∙R perianal-superior  ∙R buttock  ∙R perianal-inferior  *Size of lesion(s)*:  ∙5.5cm x 5.0cm (27.5cm^2^)  ∙4.0cm x 3.0cm (12.0cm^2^)  ∙4.0cm x 4.0cm (16.0cm^2^)  *History*  *Metastases*: None  *Prior treatment and response to treatment*:   - none | PDT:  *Skin Prep*: 1% lignocaine  *Drug*: Porfimer sodium (Photofrin)  *Dosage*: 1mg/kg  *Route of Admin*.: intravenous  *Time to Photoact.*: 48 hours  *Light Source*: argon dye laser, 632.8nm  *Light Dose*: 215 J/cm^2^  *Light Intensity*: 150 mW/cm^2^  *Treatment Time*: NR  *No. of Treatments*: 1  *note: performed in OR under general anesthesia | *Outcomes*:  Lesion response  - clinical and histological (2 biopsies) exam  -CR = 100% disease clearance, PR = 50-99% clearance, MR = < 50% clearance (minimal response)  -recurrence: clinical assessment after CR | *Outcomes*: (per lesion)  R perianal-superior  -at 6 months: CR (no retreatment)  -recurrence: none  R buttock  -at 6 months: CR (no retreatment)  -recurrence: N/A  R perianal-inferior  -at 6 months: CR (no retreatment)  -recurrence: N/A | 4 |
|  |  |  |  | Cosmetic Outcome  -clinical assessment | -greater incidence of mild scarring in pts. treated with Porfimer-sodium-PDT, but still less severe than prior surgical scars  -no functional impairments |  |
|  |  |  |  | *Adverse Events*:  -erythema and swelling; clinical examination | *Adverse Events*:  -no major adverse events  -healing in 2-3 weeks post treatment (back to work) and complete healing in 3 months |  |
|  |  | *Patient characteristics*  *Patient no.*: 8  *Gender*: female  *Age*: 52 years  *Length of Follow-up*: 48-68 months  *Lesion characteristics*  *No. of lesions*: 2  *Location of lesion(s):*  ∙perianal  *Size of lesion(s)*:  ∙6.0cm x 6.0cm (36.0cm^2^)  ∙1.0cm x 1.0cm (1.0cm^2^)  *History*  *Metastases*: None  *Prior treatment and response to treatment*:   - surgical and laser excision on both | PDT:  *Skin Prep*: 1% lignocaine  *Drug*: Porfimer sodium (Photofrin)  *Dosage*: 2mg/kg  *Route of Admin*.: intravenous  *Time to Photoact.*: 48 hours  *Light Source*: argon dye laser, 632.8nm  *Light Dose*: 215 J/cm^2^  *Light Intensity*: 150 mW/cm^2^  *Treatment Time*: NR  *No. of Treatments*: 1  *note: performed in OR under general anesthesia | *Outcomes*:  Lesion response  - clinical and histological (2 biopsies) exam  -CR = 100% disease clearance, PR = 50-99% clearance, MR = < 50% clearance (minimal response)  -recurrence: clinical assessment after CR | *Outcomes*: (per lesion)  Perianal (both)  -at 6 months: CR (no retreatment)  -recurrence: none | 4 |
|  |  |  |  | Cosmetic Outcome  -clinical assessment | -greater incidence of mild scarring in pts. treated with Porfimer-sodium-PDT, but still less severe than prior surgical scars  -no functional impairments |  |
|  |  |  |  | *Adverse Events*:  -erythema and swelling; clinical examination | *Adverse Events*:  -no major adverse events  -healing in 2-3 weeks post treatment (back to work) and complete healing in 3 months |  |
| *Notes:*  -8 patients (24 lesions) with histologically verified EMPD reviewed – 7/8 Fitzpatrick skin type I or II, 1/8 type III  -Shieh et al. (2002) provided results of 5 patients/16 lesions treated with ALA-PDT (1 lesion re-treated with Photofrin), 11 lesions had prior failed treatment  -Housel et al. (2010) provided additional follow-up data and results of an additional 3 patients/8 lesions treated with Photofrin-PDT, 5 lesions had prior failed treatment  -9 lesions treated with Photofrin-PDT (8 from Housel et al. and 1 from Shieh et al.) – 7/9 (78%) had CR at 12-96 months, 2/11 (18%) had PR and were not retreated  -**summary**:  --Shieh et al. (2002): 5 male patients with EMPD (16 lesions), mean age ± SD: 64.5 ± 9.86 years (range: 50-75), treated with ALA-PDT: 1-5 treatments (1 lesion in 1 patient also treated with 1 IV Porfimer sodium PDT treatment), for previously untreated (5/16) lesions or treatment-resistant (11/16) lesions after conventional treatment failure; results: after 1-2 treatments-CR 8/16 (50%), PR 3/16 (19%), MR 5/16 (31%); recurrence-3/8 CR lesions after 9, 10 and 10 months (1/3 recurrences had CR after retreatment with ALA-PDT); 2/3 PR had PR after repeat ALA-PDT, 1/3 PR refused additional ALA-PDT; 2/5 MR had CR after repeat ALA-PDT with recurrence at 4 years post-treatment and death of unrelated disease at 6 years follow-up; follow-up from 6-71 months  --Housel et al. (2010): 3 female patients with EMPD (8 lesions), mean age ± SD: 70.0 ± 15.6 years (range: 52-80), treated with Porfimer-Sodium-PDT: 1 treatment, for previously untreated (3/8) lesions or treatment-resistant (5/8) lesions after conventional treatment failure; results: after 1 treatment-CR 6/8 (75%), PR 2/8 (25%); no recurrence, no retreatment | | | | | | |
| - **Thaler et al. (2010)** - Austria - EMPD | *Design*:  retrospective  case report  *Setting*:  single-centre  *Number of patients*: 1 | *Patient characteristics*  *Patient no.*: 1  *Gender*: male  *Age*: 69 years  *Length of Follow-up*: NR  *Lesion characteristics*  *No. of lesions*: 1  *Location of lesion(s):* perianal  *Size of lesion(s)*:  NR  *History*  *Metastases*: NR  *Prior treatment and response to treatment*:   - NR | PDT:  *Skin Prep*: NR  *Drug*: 20% 5-ALA  *Dosage*: NR  *Route of Admin*.: topical  *Time to Photoact.*: 5 hours  *Light Source*: Woods lamp  *Light Dose*: 120 J/cm^2^  *Light Intensity*: NR  *Treatment Time*: NR  *No. of Treatment:* 4 | *Outcomes*:  Lesion response  -clinical and histological exam | *Outcomes :*  -after 4 treatments : PR  "good macroscopic clinical result" but biopsy showed tumour cells | 4 |
|  |  |  |  | Cosmetic Outcome  -patient-reported | -patient reported “a very satisfactory response” and that “for the first time in weeks he was able to sit again” |  |
|  |  |  |  | *Adverse Events*:  -NR | *Adverse Events*:  -NR |  |
| *Notes:*  -after first treatment patient reported a satisfactory response: “for the first time in weeks he was able to sit again”  -**summary**: 1 patient with EMPD (1 lesion) age 69 years treated with 4*ALA-PDT; results: after 4 treatments-PR 1/1 (100%) | | | | | | |
| - **Andretta-Tanaka et al. (2009)** - Brazil - EMPD | *Design*: retrospective case series  *Setting*: single-centre, academic clinic  *Number of patients:* 4 | *Patient characteristics*  *Patient no.*: 1  *Gender*: male  *Age*: 68 years  *Length of Follow-up*: 12 months  *Lesion characteristics*  *No. of lesions*: 1  *Location of lesion(s):* axilla  *Size of lesion(s)*: 8cm diam.  *History*  *Metastases*: NR  *Prior treatment and response to treatment*:   - previously treated in other hospitals with anti-fungal’s and corticosteroids 🡪 no therapeutic response - topical imiquimod (5%) cream 5 times a week for 6 months 🡪 lesion decreased in size by approx. 50% | PDT:  *Skin Prep*: cleansed with 0.9% saline  *Drug*: 16% m-ALA (Metvix®)  *Dosage*: applied on lesion + 1cm clinically disease-free margin  *Route of Admin*.: topical  *Time to Photoact.*: 3 hours  *Light Source*: visible red light, 630nm  *Light Dose*: 37 J/cm^2^  *Light Intensity*: NR  *Treatment Time*: NR  *No. of Treatments*: 8 total – 3 (2 weeks apart) + 5 more after biopsy revealed neoplastic cells | *Outcomes*:  Lesion response  -clinical and histological (biopsy) exam | *Outcomes*:  -after 3 treatments: PR  -after 5 more treatments: PR  (lesion decreased in size by 60%) | 4 |
|  |  |  |  | *Adverse Events*:  NR | *Adverse Events*:  NR |  |
|  |  | *Patient characteristics*  *Patient no.*: 2  *Gender*: male  *Age*: 76 years  *Length of Follow-up*: 6 months  *Lesion characteristics*  *No. of lesions*: 1  *Location of lesion(s):* scrotum  *Size of lesion(s)*: 5cm diam.  *History*  *Metastases*: NR  *Prior treatment and response to treatment*:   - previously treated in other hospitals with anti-fungal’s and corticosteroids 🡪 no therapeutic response | PDT:  *Skin Prep*: cleansed with 0.9% saline  *Drug*: 16% m-ALA (Metvix®)  *Dosage*: applied on lesion + 1cm clinically disease-free margin  *Route of Admin*.: topical  *Time to Photoact.*: 3 hours  *Light Source*: visible red light, 630nm  *Light Dose*: 37 J/cm^2^  *Light Intensity*: NR  *Treatment Time*: NR  *No. of Treatments*: 3 total – 3 (2 weeks apart) | *Outcomes*:  Lesion response  -clinical and histological (biopsy) exam | *Outcomes*:  -after 3 treatments: CR |  |
|  |  |  |  | *Adverse Events*:  NR | *Adverse Events*:  NR |  |
|  |  | *Patient characteristics*  *Patient no.*: 3  *Gender*: female  *Age*: 73 years  *Length of Follow-up*: 6 months  *Lesion characteristics*  *No. of lesions*: 1  *Location of lesion(s):* vulva  *Size of lesion(s)*: 10cm diam.  *History*  *Metastases*: NR  *Prior treatment and response to treatment*:   - previously treated in other hospitals with anti-fungal’s and corticosteroids 🡪 no therapeutic response | PDT:  *Skin Prep*: cleansed with 0.9% saline  *Drug*: 16% m-ALA (Metvix®)  *Dosage*: applied on lesion + 1cm clinically disease-free margin  *Route of Admin*.: topical  *Time to Photoact.*: 3 hours  *Light Source*: visible red light, 630nm  *Light Dose*: 37 J/cm^2^  *Light Intensity*: NR  *Treatment Time*: NR  *No. of Treatments*: 3 total – 3 (2 weeks apart) + plans for 3 more treatments in future | *Outcomes*:  Lesion response  -clinical and histological (biopsy) exam | *Outcomes*:  -after 3 treatments: PR  (lesion decreased in size by 40-60%) |  |
|  |  |  |  | Patient Comfort and QoL  -reported by patient | -improved comfort level  -improved QOL |  |
|  |  |  |  | *Adverse Events*:  NR | *Adverse Events*:  NR |  |
|  |  | *Patient characteristics*  *Patient no.*: 4  *Gender*: female  *Age*: 67 years  *Length of Follow-up*: 6 months  *Lesion characteristics*  *No. of lesions*: 1  *Location of lesion(s):* vulva  *Size of lesion(s)*: 20cm diam.  *History*  *Metastases*: NR  *Prior treatment and response to treatment*:   - previously treated in other hospitals with anti-fungal’s and corticosteroids 🡪 no therapeutic response | PDT:  *Skin Prep*: cleansed with 0.9% saline  *Drug*: 16% m-ALA (Metvix®)  *Dosage*: applied on lesion + 1cm clinically disease-free margin  *Route of Admin*.: topical  *Time to Photoact.*: 3 hours  *Light Source*: visible red light, 630nm  *Light Dose*: 37 J/cm^2^  *Light Intensity*: NR  *Treatment Time*: NR  *No. of Treatments*: 3 total – 3 (2 weeks apart) + plans for 3 more treatments in future | *Outcomes*:  Lesion response  -clinical and histological (biopsy) exam | *Outcomes*:  -after 3 treatments: PR  (lesion decreased in size by 40-60%) |  |
|  |  |  |  | Patient Comfort and QoL  -reported by patient | -improved comfort level  -improved QOL |  |
|  |  |  |  | *Adverse Events*:  NR | *Adverse Events*:  NR |  |
| *Notes:*  -4 patients described above (treated with PDT) were part of a group of 14 patients diagnosed with Paget’s disease at a clinic in Brazil (and treated with other modalities)  -**summary**: 4 patients with EMPD (4 lesions total), mean age ± SD: 71 ± 4 years (range: 67-76), treated with MAL-PDT: 3-8 treatments (2 weeks apart); results: after 3 treatments-CR 1/4 (25%) patients, PR-3/4 (75%) patients; follow-up from 6-12 months | | | | | | |
| - **Fukui et al. (2009)** - Japan - EMPD | *Design*:  retrospective  case series  *Setting*:  single-centre,  academic clinic  *Number of patients*: 5 | *Patient characteristics*  *Patient no.*: 1  *Gender*: male  *Age*: 81 years  *Length of Follow-up*: 18 months  *Lesion characteristics*  *No. of lesions*: 1  *Location of lesion(s):* penile base  *Size of lesion(s)*: NR  *History*  *Metastases*: NR  *Prior treatment and response to treatment*:   - ALA-PDT 🡪 failed | PDT:  *Skin Prep*: local anesthesia (1% xylocaine); CO_2_ laser treatment 1 cm away from lesion  *Drug*: 20% 5-ALA  *Dosage*: NR  *Route of Admin*.: topical  *Time to Photoact.*: 3 hours  *Light Source*: pulsed (excimer) dye laser, 630nm (PDT EDL-1)  *Light Dose*: 300 J/cm^2^ (3 x 100J/cm^2^ per treatment session)  *Light Intensity*: NR  *Treatment Time*: NR  *No. of Treatments*: 2 treatment cycles with each cycle = 3 irradiation sessions 2 weeks apart (pt. received a total of 630 J/cm^2^ of ALA-PDT) | *Outcomes*:  Lesion response  -clinical and histological (biopsy) exam | *Outcomes*:  -after 2 treatments: CR | 4 |
|  |  |  |  | *Adverse Events*:  NR | *Adverse Events*:  NR |  |
|  |  | *Patient characteristics*  *Patient no.*: 2  *Gender*: female  *Age*: 84 years  *Length of Follow-up*: 17 months  *Lesion characteristics*  *No. of lesions*: 1  *Location of lesion(s):* labia majora  *Size of lesion(s)*: NR  *History*  *Metastases*: NR  *Prior treatment and response to treatment*:   - ALA-PDT 🡪 failed | PDT:  *Skin Prep*: local anesthesia (1% xylocaine); CO_2_ laser treatment 1cm away from lesion  *Drug*: 20% 5-ALA  *Dosage*: NR  *Route of Admin*.: topical  *Time to Photoact.*: 3 hours  *Light Source*: pulsed (excimer) dye laser, 630nm (PDT EDL-1)  *Light Dose*: 300 J/cm^2^ (3 x 100J/cm^2^ per treatment session)  *Light Intensity*: NR  *Treatment Time*: NR  *No. of Treatments*: 3 treatment cycles with each cycle = 3 irradiation sessions 2 weeks apart (pt. received a total of 1050 J/cm^2^ of ALA-PDT) | *Outcomes*:  Lesion response  -clinical and histological (biopsy) exam | *Outcomes*:  -after 3 treatments: CR (in 2 of 4 areas*) |  |
|  |  |  |  | *Adverse Events*:  NR | *Adverse Events*:  NR |  |
|  |  | *Patient characteristics*  *Patient no.*: 3  *Gender*: female  *Age*: 66 years  *Length of Follow-up*: 15 months  *Lesion characteristics*  *No. of lesions*: 2  *Location of lesion(s):* vulva & perianal  *Size of lesion(s)*: NR  *History*  *Metastases*: NR  *Prior treatment and response to treatment*:   - excision 🡪 residual tumor present following excision | PDT:  *Skin Prep*: local anesthesia (1% xylocaine); CO_2_ laser treatment 1cm away from lesion  *Drug*: 20% 5-ALA  *Dosage*: NR  *Route of Admin*.: topical  *Time to Photoact.*: 3 hours  *Light Source*: pulsed (excimer) dye laser, 630nm (PDT EDL-1)  *Light Dose*: 300 J/cm^2^ (3 x 100J/cm^2^ per treatment session)  *Light Intensity*: NR  *Treatment Time*: NR  *No. of Treatments*: 3 treatment cycles with each cycle = 3 irradiation sessions 2 weeks apart | *Outcomes*:  Lesion response  -clinical and histological (biopsy) exam | *Outcomes*:  -after 3 treatments: CR |  |
|  |  |  |  | *Adverse Events*:  NR | *Adverse Events*:  NR |  |
|  |  | *Patient characteristics*  *Patient no.*: 4  *Gender*: female  *Age*: 76 years  *Length of Follow-up*: 12 months  *Lesion characteristics*  *No. of lesions*: 2  *Location of lesion(s):* vulva & extra-urethra  *Size of lesion(s)*: NR  *History*  *Metastases*: NR  *Prior treatment and response to treatment*:   - excision 🡪 residual tumor present following excision | PDT:  *Skin Prep*: local anesthesia (1% xylocaine); CO_2_ laser treatment 0.5cm away from lesion  *Drug*: 20% 5-ALA  *Dosage*: NR  *Route of Admin*.: topical  *Time to Photoact.*: 3 hours  *Light Source*: pulsed (excimer) dye laser, 630nm (PDT EDL-1)  *Light Dose*: 300 J/cm^2^ (3 x 100J/cm^2^ per treatment session)  *Light Intensity*: NR  *Treatment Time*: NR  *No. of Treatments*: NR | *Outcomes*:  Lesion response  -clinical and histological (biopsy) exam | *Outcomes*:  -after ? treatment(s): CR |  |
|  |  |  |  | *Adverse Events*:  NR | *Adverse Events*:  NR |  |
|  |  | *Patient characteristics*  *Patient no.*: 5  *Gender*: female  *Age*: 83 years  *Length of Follow-up*: 3 months**  *Lesion characteristics*  *No. of lesions*: 2  *Location of lesion(s):* vulva & labia majora  *Size of lesion(s)*: NR  *History*  *Metastases*: NR  *Prior treatment and response to treatment*:   - excision 🡪 residual tumor present following excision | PDT:  *Skin Prep*: local anesthesia (1% xylocaine); CO_2_ laser treatment 1 cm away from lesion  *Drug*: 20% 5-ALA  *Dosage*: NR  *Route of Admin*.: topical  *Time to Photoact.*: 3 hours  *Light Source*: pulsed (excimer) dye laser, 630nm (PDT EDL-1)  *Light Dose*: 300 J/cm^2^ (3 x 100J/cm^2^ per treatment session)  *Light Intensity*: NR  *Treatment Time*: NR  *No. of Treatments*: NR | *Outcomes*:  Lesion response  -clinical and histological (biopsy) exam | *Outcomes*:  -after ? treatment(s): CR |  |
|  |  |  |  | *Adverse Events*:  NR | *Adverse Events*:  NR |  |
| *Notes:*  -all patients received CO_2_ laser treatment prior to ALA-PDT (to improve PDT permeability)  -*in patient 2, lesion was divided into 4 areas, 3 of which were treated and the other served as non-treated control; complete response was reported in 2 of the treated areas  -**patient 5 showed CR at 3 months but died of other unrelated causes (intracranial hemorrhage)  -**summary**: 5 patients with EMPD (8 lesions total), mean age ± SD: 78 ± 7.4 years (range: 66-84), treated with ALA-PDT: 2-3 treatments (each treatment = 3 sessions, 2 weeks apart) after prior failed treatments (2 with ALA-PDT and 3 with surgical excision); results: after 2-3 PDT treatments-CR 5/5 (100%) patients; follow-up from 3-18 months | | | | | | |
| - **Li et al. (2009)** - China - EMPD | *Design*:  retrospective  case report  *Setting*:  Single-centre, academic clinic  *Number of patients*: 1 | *Patient characteristics*  *Patient no.*: 1  *Gender*: female  *Age*: 75 years  *Length of Follow-up*: 7 months  *Lesion characteristics*  *No. of lesions*: 1  *Location of lesion(s):* perianal  *Size of lesion(s)*:  2.5cm x 2.5cm (6.25 cm^2^)  *History*  *Metastases*: None  *Prior treatment and response to treatment*:   - hemorrhoid previously treated with surgery - pt. declined further surgery | PDT:  Treatment 1  *Skin Prep*: NR  *Drug*: 20% 5-ALA  *Dosage*: applied on lesion + 2cm clinically disease-free margin  *Route of Admin*.: topical  *Time to Photoact.*: 3 hours  *Light Source*: diode laser (630 PDT laser, Diomed), 630nm  *Light Dose*: 120 J/cm^2^  *Light Intensity*: NR  *Treatment Time*: NR  *No. of Treatment:* 2 (1 day apart)  Treatment 2  *Skin Prep*: NR  *Drug*:  ∙ 20% 5-ALA  ∙ Hematoporphyrin (HpD)  *Dosage*:  ∙ applied on lesion + 2cm clinically disease-free margin  ∙ 5mg/kg  *Route of Admin*.:  ∙ topical  ∙intravenous  *Time to Photoact.*:  ∙ 3 hours  ∙ 48 hours  *Light Source*: diode laser (630 PDT laser, Diomed), 630nm  *Light Dose*: 120 J/cm^2^  *Light Intensity*: NR  *Treatment Time*: NR  *No. of Treatments*: 1 | *Outcomes*:  Lesion response  -clinical and histological (biopsy) exam | *Outcomes*:  -after 3 treatments: CR | 4 |
|  |  |  |  | Cosmetic outcome  -assessed by investigators | -preserved anus and anal function, free of scarring |  |
|  |  |  |  | Patient satisfaction  -report by investigators | -author reports “patient was satisfied” |  |
|  |  |  |  | *Adverse Events*:  NR | *Adverse Events*:  NR |  |
| *Notes:*  -treatment 1: patient had 2 courses of topical 5-ALA-PDT within 2 days (due to residual lesion after first treatment)  -treatment 2: a third course of “more rigorous” PDT treatment took place at 40 days after initial ALA-PDT due to residual lesion; this treatment combined topical ALA-PDT and IV Hematoporphyrin  -**summary**: 1 patient with EMPD (1 lesion) age 75 years treated with ALA-PDT: 2 treatments (1 day apart) + combined topical ALA-PDT + IV Hematoporphyrin -PDT (40 days later); results: after 3 treatments-CR 1/1 (100%) patient (disease free at 7 months after last treatment) | | | | | | |
| - **Wang et al. (2008)** - China - EMPD*,* MPD | *Design*:  retrospective case series  *Setting*:  single-centre, general clinic  *Number of patients*:4  (3 EMPD,  1 MPD) | *Patient characteristics*  *Patient no.*: 1  *Gender*: male  *Age*: 66 years  *Length of Follow-up*: NR  *Lesion characteristics*  *No. of lesions*: 1  *Location of lesion(s):* penis/scrotum/mons pubis  *Size of lesion(s)*:  6cm x 5cm (30cm^2^) and  9cm x 5cm (45cm^2^)  *History*  *Metastases*: NR  *Prior treatment and response to treatment*:   - cryotherapy 🡪 “poor response” | PDT:  *Skin Prep*: acetone and NaCl cleanse + scab removal  *Drug*: 5-ALA (20% cream or 10% solution)  *Dosage*: NR  *Route of Admin*.: topical  *Time to Photoact.*: 3-5 hours  *Light Source*: He-Ne laser or diode laser, 635nm  *Light Dose*: NR  *Light Intensity*: 60 mW/cm^2^  *Treatment Time*: NR  *No. of Treatments*: 3 | *Outcomes*:  Lesion response  -clinical and histological (biopsy) exam | *Outcomes*:  -after 3 treatments: PR  (lesion size reduction)  -patient eventually underwent surgery as PDT did not achieve “complete cure” | 4 |
|  |  |  |  | *Adverse Events*:  Not reported by disease/case specific reports | *Adverse Events*:  NR |  |
|  |  | *Patient characteristics*  *Patient no.*: 2  *Gender*: NR  *Age*: NR  *Length of Follow-up*: 12 months  *Lesion characteristics*  *No. of lesions*: NR  *Location of lesion(s):* NR  *Size of lesion(s)*: NR  *History*  *Metastases*: NR  *Prior treatment and response to treatment*:   - NR | PDT:  *Skin Prep*: acetone and NaCl cleanse + scab removal  *Drug*: 5-ALA (20% cream or 10% solution)  *Dosage*: NR  *Route of Admin*.: topical  *Time to Photoact.*: 3-5 hours  *Light Source*: He-Ne laser or diode laser, 635nm  *Light Dose*: NR  *Light Intensity*: 60 mW/cm^2^  *Treatment Time*: NR  *No. of Treatments*: NR | *Outcomes*:  Lesion response  -clinical and histological (biopsy) exam | *Outcomes*:  -after ? treatment(s): PR  (lesions size reduction)  -recurrence within 1 year of follow-up |  |
|  |  |  |  | *Adverse Events*:  Not reported by disease/case specific reports | *Adverse Events*:  NR |  |
|  |  | *Patient characteristics*  *Patient no.*: 3  *Gender*: NR  *Age*: NR  *Length of Follow-up*: 12 months  *Lesion characteristics*  *No. of lesions*: NR  *Location of lesion(s):* NR  *Size of lesion(s)*: NR  *History*  *Metastases*: NR  *Prior treatment and response to treatment*:   - NR | PDT:  *Skin Prep*: acetone and NaCl cleanse + scab removal  *Drug*: 5-ALA (20% cream or 10% solution)  *Dosage*: NR  *Route of Admin*.: topical  *Time to Photoact.*: 3-5 hours  *Light Source*: He-Ne laser or diode laser, 635nm  *Light Dose*: NR  *Light Intensity*: 60 mW/cm^2^  *Treatment Time*: NR  *No. of Treatments*: NR | *Outcomes*:  Lesion response  -clinical and histological (biopsy) exam | *Outcomes*:  -after ? treatment(s): PR  (lesions size reduction)  -recurrence within 1 year of follow-up |  |
|  |  |  |  | *Adverse Events*:  Not reported by disease/case specific reports | *Adverse Events*:  NR |  |
|  |  | *Patient characteristics*  *Patient no.*: 4  *Gender*: female  *Age*: 28 years  *Length of Follow-up*: 12 months  *Lesion characteristics*  *No. of lesions*: NR  *Location of lesion(s):* mammary  *Size of lesion(s)*: NR  *History*  *Metastases*: NR  *Prior treatment and response to treatment*:   - radical mastectomy 🡪 recurrence at 4 months | PDT:  *Skin Prep*: acetone and NaCl cleanse + scab removal  *Drug*: 5-ALA (20% cream or 10% solution)  *Dosage*: NR  *Route of Admin*.: topical  *Time to Photoact.*: 3-5 hours  *Light Source*: He-Ne laser or diode laser, 635nm  *Light Dose*: NR  *Light Intensity*: 60 mW/cm^2^  *Treatment Time*: NR  *No. of Treatments*: 2 | *Outcomes*:  Lesion response  -clinical and histological (biopsy) exam | *Outcomes*:  -after 2 treatments: CR  -no recurrence within 1 year of follow-up |  |
|  |  |  |  | *Adverse Events*:  Not reported by disease/case specific reports | *Adverse Events*:  NR |  |
| *Notes:*  -4 Paget’s patients described above (treated with PDT) were part of a group of 76 skin cancer patients treated with PDT; brief case reports were provided for only 2 of the pts. (1 EMPD, 1 MPD) – specific demographic and lesion characteristics not provided for other 2 patients; specific intervention information not described per patient  -**summary**: 3 patients with EMPD and 1 patient with MPD (gender and age specific data provided for only 2 case-reports: 66 year male with EMPD and 28 year female with MPD) treated PDT (female pt. had ALA-PDT for recurrence after radical mastectomy; male pt. had ALA-PDT as primary treatment; no details on the other 2 pts. provided); results: EMPD-PR 3/3 (100%), with recurrence in 2/3 (67%) at 1 year follow-up; MPD-CR 1/1 (100%) at 1 year follow-up; overall-CR 1/4 (25%), PR 3/4 (75%), recurrence 2/4 (50%) | | | | | | |
| - **Liu et al. (2007)** - China - EMPD | *Design*:  retrospective  case series  *Setting*:  single-centre,  general clinic  *Number of patients*: 5 | *Patient characteristics*  *Patient no.*: 1  *Gender*: male  *Age*: NR  *Length of Follow-up*: 3 months  *Lesion characteristics*  *No. of lesions*: 1  *Location of lesion(s):* scrotum  *Size of lesion(s)*: NR  *History*  *Metastases*: NR  *Prior treatment and response to treatment*:   - NR | PDT:  *Skin Prep*: NR  *Drug*: Photofrin  *Dosage*: 2mg/kg  *Route of Admin*.: intravenous  *Time to Photoact.*: 24 hours  *Light Source*: 630-PDT diode laser (Diomed)  *Light Dose*: 150-300 J/cm^2^  *Light Intensity*: 100-150mW/cm^2^  *Treatment Time*: NR  *No. of Treatments*: 1 treatment cycle (each with 3 sessions within 72 hours of photosensitizer administration) | *Outcomes*:  No patient specific data reported | *Outcomes*:  NR | 4 |
|  |  |  |  | *Adverse Events*: | *Adverse Events*:  -authors report: “no severe complications”  -blistering and effusion 48 hours after irradiation, scar formation 96-120 hours later |  |
|  |  | *Patient characteristics*  *Patient no.*: 2  *Gender*: male  *Age*: NR  *Length of Follow-up*: 3 months  *Lesion characteristics*  *No. of lesions*: 1  *Location of lesion(s):* radix penis  *Size of lesion(s)*: NR  *History*  *Metastases*: NR  *Prior treatment and response to treatment*:   - NR | PDT:  *Skin Prep*: NR  *Drug*: Photofrin  *Dosage*: 2mg/kg  *Route of Admin*.: intravenous  *Time to Photoact.*: 24 hours  *Light Source*: 630-PDT diode laser (Diomed)  *Light Dose*: 150-300 J/cm^2^  *Light Intensity*: 100-150mW/cm^2^  *Treatment Time*: NR  *No. of Treatments*: 1 treatment cycle (each with 3 sessions within 72 hours of photosensitizer administration) | *Outcomes*:  No patient specific data reported | *Outcomes*:  NR |  |
|  |  |  |  | *Adverse Events*: | *Adverse Events*:  -authors report: “no severe complications”  -blistering and effusion 48 hours after irradiation, scar formation 96-120 hours later |  |
|  |  | *Patient characteristics*  *Patient no.*: 3  *Gender*: male  *Age*: NR  *Length of Follow-up*: 3 months  *Lesion characteristics*  *No. of lesions*: 1  *Location of lesion(s):* crissum  *Size of lesion(s)*: NR  *History*  *Metastases*: NR  *Prior treatment and response to treatment*:   - NR | PDT:  *Skin Prep*: NR  *Drug*: Photofrin  *Dosage*: 2mg/kg  *Route of Admin*.: intravenous  *Time to Photoact.*: 24 hours  *Light Source*: 630-PDT diode laser (Diomed)  *Light Dose*: 150-300 J/cm^2^  *Light Intensity*: 100-150mW/cm^2^  *Treatment Time*: NR  *No. of Treatments*: 1 treatment cycle (each with 3 sessions within 72 hours of photosensitizer administration) | *Outcomes*:  No patient specific data reported | *Outcomes*:  NR |  |
|  |  |  |  | *Adverse Events*: | *Adverse Events*:  -authors report: “no severe complications”  -blistering and effusion 48 hours after irradiation, scar formation 96-120 hours later |  |
|  |  | *Patient characteristics*  *Patient no.*: 4  *Gender*: female  *Age*: NR  *Length of Follow-up*: 3 months  *Lesion characteristics*  *No. of lesions*: 1  *Location of lesion(s):* mons veneris  *Size of lesion(s)*: NR  *History*  *Metastases*: NR  *Prior treatment and response to treatment*:   - NR | PDT:  *Skin Prep*: NR  *Drug*: Photofrin  *Dosage*: 2mg/kg  *Route of Admin*.: intravenous  *Time to Photoact.*: 24 hours  *Light Source*: 630-PDT diode laser (Diomed)  *Light Dose*: 150-300 J/cm^2^  *Light Intensity*: 100-150mW/cm^2^  *Treatment Time*: NR  *No. of Treatments*: 1 treatment cycle (each with 3 sessions within 72 hours of photosensitizer administration) | *Outcomes*:  No patient specific data reported | *Outcomes*:  NR |  |
|  |  |  |  | *Adverse Events*: | *Adverse Events*:  -authors report: “no severe complications”  -blistering and effusion 48 hours after irradiation, scar formation 96-120 hours later |  |
|  |  | *Patient characteristics*  *Patient no.*: 5  *Gender*: female  *Age*: NR  *Length of Follow-up*: 3 months  *Lesion characteristics*  *No. of lesions*: 1  *Location of lesion(s):* labium majus  *Size of lesion(s)*: NR  *History*  *Metastases*: NR  *Prior treatment and response to treatment*:   - NR | PDT:  *Skin Prep*: NR  *Drug*: Photofrin  *Dosage*: 2mg/kg  *Route of Admin*.: intravenous  *Time to Photoact.*: 24 hours  *Light Source*: 630-PDT diode laser (Diomed)  *Light Dose*: 150-300 J/cm^2^  *Light Intensity*: 100-150mW/cm^2^  *Treatment Time*: NR  *No. of Treatments*: 1 treatment cycle (each with 3 sessions within 72 hours of photosensitizer administration) | *Outcomes*:  No patient specific data reported | *Outcomes*:  NR |  |
|  |  |  |  | *Adverse Events*: | *Adverse Events*:  -authors report: “no severe complications”  -blistering and effusion 48 hours after irradiation, scar formation 96-120 hours later |  |
| *Notes:*  -demographic information not provided per patient (only pt. population overall)  -**summary**: 5 patients with EMPD (5 lesions total), mean age: 74.6 years (range: 64-80 years) treated with PDT; 2 pts. initially treated with corticosteroids and topical antibiotics 🡪 refused surgery; 3 pts. prior failed treatments (1 with 3 surgical excisions 🡪 relapsed after radiotherapy + local chemotherapy; 1 with CO_2_ laser 🡪 relapsed and refused surgery; 1 with local chemotherapy 🡪 no response); results: after 1 treatment-CR 1/5 (20%) patients, PR-3/5 (60%) patients (50% reduction in lesion size), and 1/5 (20%) showed “slight shrinking of lesion” at 3 months follow-up | | | | | | |
| - **T’Kint & Roseeuw (2006)** - Belgium - EMPD | *Design*:  retrospective  case report  *Setting*:  single-centre,  academic clinic  *Number of patients*:1 | *Patient characteristics*  *Patient no.*: 1  *Gender*: female  *Age*: 64 years  *Length of Follow-up*: 14 months  *Lesion characteristics*  *No. of lesions*: 2*  *Location of lesion(s):*  perivulvar region  perianal region  *Size of lesion(s)*: NR  *History*  *Metastases*: None  *Prior treatment and response to treatment*:   - PDT used as adjunct therapy with wide local surgical excision and VY plasty | PDT:  *Skin Prep*: NR  *Drug*: m-ALA  *Dosage*: NR  *Route of Admin*.: topical  *Time to Photoact.*: NR  *Light Source*: red light  *Light Dose*: NR  *Light Intensity*: NR  *Treatment Time*: NR  *No. of Treatments*: 4 (2 weeks apart) | *Outcomes*:  Lesion response  -histological (biopsy) exam | *Outcomes*:  -after 4 treatments (at 14 months follow-up): CR | 4 |
|  |  |  |  | *Adverse Events*:  NR | *Adverse Events*:  NR |  |
| *Notes:*  -*unclear if there is one lesion of the perivulvar/perianal region or 2 lesions-1 perivulvar and 1 perianal  -MAL-PDT used as adjunct with Mohs Micrographic surgery to preserve cosmetic and functional aspects of the region  -**summary**: 1 female patient with EMPD (2 lesions total) age 64 years treated with MAL-PDT: 4 treatments (2 weeks apart) as adjunct with Mohs surgery for functional and cosmetic purposes; results: after 4 treatments-CR 2/2 (100%), with no recurrence after 14 months of follow-up | | | | | | |
| - **Kim et al. (2005)** - Korea - EMPD | *Design*:  retrospective  case series  *Setting*:  single-centre,  academic clinic  *Number of patients*:7 | *Patient characteristics*  *Patient no.*: 1  *Gender*: male  *Age*: 54 years  *Length of Follow-up*: NR  *Lesion characteristics*  *No. of lesions*: NR  *Location of lesion(s):*  scrotum  *Size of lesion(s)*:  NR  *History*  *Metastases*: no  *Prior treatment and response to treatment*:   - NR | PDT:  *Skin Prep*: NR  *Drug*: NR  *Dosage*: NR  *Route of Admin*.: NR  *Time to Photoact.*: NR  *Light Source*: NR  *Light Dose*: NR  *Light Intensity*: NR  *Treatment Time*: NR  *No. of Treatments*: NR | *Outcomes*:  Lesion response  -NR | *Outcomes*:  -no recurrence | 4 |
|  |  |  |  | *Adverse Events*:  -NR | *Adverse Events*:  -NR |  |
|  |  | *Patient characteristics*  *Patient no.*: 2  *Gender*: male  *Age*: 69 years  *Length of Follow-up*: NR  *Lesion characteristics*  *No. of lesions*: NR  *Location of lesion(s):*  penis/scrotum  *Size of lesion(s)*:  NR  *History*  *Metastases*: no  *Prior treatment and response to treatment*:   - NR | PDT:  *Skin Prep*: NR  *Drug*: NR  *Dosage*: NR  *Route of Admin*.: NR  *Time to Photoact.*: NR  *Light Source*: NR  *Light Dose*: NR  *Light Intensity*: NR  *Treatment Time*: NR  *No. of Treatments*: NR | *Outcomes*:  Lesion response  -NR | *Outcomes*:  -no recurrence |  |
|  |  |  |  | *Adverse Events*:  -NR | *Adverse Events*:  -NR |  |
|  |  | *Patient characteristics*  *Patient no.*: 3  *Gender*: male  *Age*: 56 years  *Length of Follow-up*: NR  *Lesion characteristics*  *No. of lesions*: NR  *Location of lesion(s):*  scrotum  *Size of lesion(s)*:  NR  *History*  *Metastases*: no  *Prior treatment and response to treatment*:   - NR | PDT:  *Skin Prep*: NR  *Drug*: NR  *Dosage*: NR  *Route of Admin*.: NR  *Time to Photoact.*: NR  *Light Source*: NR  *Light Dose*: NR  *Light Intensity*: NR  *Treatment Time*: NR  *No. of Treatments*: NR | *Outcomes*:  Lesion response  -NR | *Outcomes*:  -no recurrence |  |
|  |  |  |  | *Adverse Events*:  -NR | *Adverse Events*:  -NR |  |
|  |  | *Patient characteristics*  *Patient no.*: 4  *Gender*: male  *Age*: 73 years  *Length of Follow-up*: NR  *Lesion characteristics*  *No. of lesions*: NR  *Location of lesion(s):*  penis/scrotum  *Size of lesion(s)*:  NR  *History*  *Metastases*: no  *Prior treatment and response to treatment*:   - NR | PDT:  *Skin Prep*: NR  *Drug*: NR  *Dosage*: NR  *Route of Admin*.: NR  *Time to Photoact.*: NR  *Light Source*: NR  *Light Dose*: NR  *Light Intensity*: NR  *Treatment Time*: NR  *No. of Treatments*: NR | *Outcomes*:  Lesion response  -NR | *Outcomes*:  -no recurrence |  |
|  |  |  |  | *Adverse Events*:  -NR | *Adverse Events*:  -NR |  |
|  |  | *Patient characteristics*  *Patient no.*: 5  *Gender*: female  *Age*: 58 years  *Length of Follow-up*: NR  *Lesion characteristics*  *No. of lesions*: NR  *Location of lesion(s):*  vulva  *Size of lesion(s)*:  NR  *History*  *Metastases*: no  *Prior treatment and response to treatment*:   - NR | PDT:  *Skin Prep*: NR  *Drug*: NR  *Dosage*: NR  *Route of Admin*.: NR  *Time to Photoact.*: NR  *Light Source*: NR  *Light Dose*: NR  *Light Intensity*: NR  *Treatment Time*: NR  *No. of Treatments*: NR | *Outcomes*:  Lesion response  -NR | *Outcomes*:  -no recurrence |  |
|  |  |  |  | *Adverse Events*:  -NR | *Adverse Events*:  -NR |  |
|  |  | *Patient characteristics*  *Patient no.*: 6  *Gender*: male  *Age*: 54 years  *Length of Follow-up*: NR  *Lesion characteristics*  *No. of lesions*: NR  *Location of lesion(s):*  penis/scrotum  *Size of lesion(s)*:  NR  *History*  *Metastases*: no  *Prior treatment and response to treatment*:   - NR | PDT:  *Skin Prep*: NR  *Drug*: NR  *Dosage*: NR  *Route of Admin*.: NR  *Time to Photoact.*: NR  *Light Source*: NR  *Light Dose*: NR  *Light Intensity*: NR  *Treatment Time*: NR  *No. of Treatments*: NR | *Outcomes*:  Lesion response  -NR | *Outcomes*:  -no recurrence |  |
|  |  |  |  | *Adverse Events*:  -NR | *Adverse Events*:  -NR |  |
|  |  | *Patient characteristics*  *Patient no.*: 7  *Gender*: male  *Age*: 50 years  *Length of Follow-up*: NR  *Lesion characteristics*  *No. of lesions*: NR  *Location of lesion(s):*  penis/scrotum  *Size of lesion(s)*:  NR  *History*  *Metastases*: no  *Prior treatment and response to treatment*:   - NR | PDT:  *Skin Prep*: NR  *Drug*: NR  *Dosage*: NR  *Route of Admin*.: NR  *Time to Photoact.*: NR  *Light Source*: NR  *Light Dose*: NR  *Light Intensity*: NR  *Treatment Time*: NR  *No. of Treatments*: NR | *Outcomes*:  Lesion response  -NR | *Outcomes*:  -no recurrence |  |
|  |  |  |  | *Adverse Events*:  -NR | *Adverse Events*:  -NR |  |
| *Notes:*  -only abstract and table available in English (NR refers to not reported in the abstract or table)  -7 patients described above (treated with PDT & excision – order not reported) were part of a group of 28 patients diagnosed with Paget’s disease at a clinic in Korea (and treated with other modalities)  -**summary**: 7 patients with EMPD, mean age ± SD: 59.1 ± 8.5 years (range: 54-73), treated with PDT and surgical excision; results: no (0/7) recurrence | | | | | | |
| - **Madan et al. (2005)** - UK - EMPD | *Design*:  retrospective  case report  *Setting*:  single-centre,  academic clinic  *Number of patients*:1 | *Patient characteristics*  *Patient no.*: 1  *Gender*: male  *Age*: 80 years  *Length of Follow-up*: 12 months  *Lesion characteristics*  *No. of lesions*:1  *Location of lesion(s):*  left groin/scrotum  *Size of lesion(s)*:  10cm x 10cm (100cm^2^)  *History*  *Metastases*: yes (adenocarcinoma of the prostate)  *Prior treatment and response to treatment*:   - none for EMPD (patient did receive radical radiotherapy for prostatic cancer) - surgery deemed inappropriate | PDT:  Treatment 1  *Skin Prep*: 0.5% bupivacaine (for pain)  *Drug*: 20% δ 5-ALA  *Dosage*: applied on lesion + 10% margin  *Route of Admin*.: topical  *Time to Photoact.*: 6 hours  *Light Source*: red light (xenon-arc lamp), 630nm  *Light Dose*: 100 J/cm^2^  *Light Intensity*: 47-84 mW/cm^2^  *Treatment Time*: 20-35 minutes  *No. of Treatments*: 5  Treatment 2  *Skin Prep*: NR  *Drug*: Porfimer sodium (Photofrin)  *Dosage*: 1 mg/kg  *Route of Admin*.: intravenous  *Time to Photoact.*: 48 hours  *Light Source*: red light (xenon-arc lamp), 630nm  *Light Dose*: 100 J/cm^2^  *Light Intensity*: NR  *Treatment Time*: NR  *No. of Treatments*: 1  Treatment 3  *Skin Prep*: 0.5% bupivacaine (for pain)  *Drug*: 20% δ 5-ALA  *Dosage*: applied on lesion + 10% margin  *Route of Admin*.: topical  *Time to Photoact.*: 6 hours  *Light Source*: red light (xenon-arc lamp), 630nm  *Light Dose*: 100 J/cm^2^  *Light Intensity*: 47-84 mW/cm^2^  *Treatment Time*: 20-35 minutes  *No. of Treatments*: 1 | *Outcomes*:  Lesion response  -clinical and ultrasound exam | *Outcomes*:  Treatment 1  -after 1 treatment: PR (decreased ulceration and size of lesion)  -after 5 treatments: CR  -tumour recurrence at 9 months after 5^th^ treatment  Treatment 2  -CR  -local recurrence lead to treatment #3 (no time to recurrence reported)  Treatment 3  -after last treatment: CR (at 1 year follow-up) | 4 |
|  |  |  |  | *Adverse Events*:  -pain upon irradiation suspected by clinicians, post-treatment photosensitivity suspected by clinicians, inflammation post-treatment clinically observed | *Adverse Events*:  -pain: pre-treatment with 0.5% bupivacaine  -photosensitivity: post-treatment with hydrocortisone cream  -inflammation: subsided without treatment within 3 days post-treatment |  |
| *Notes:*  -patient also had a T2 ACC prostate cancer treated with radical radiotherapy  -tumour recurrence at 9 months after the last topical PDT resulted in IV Porfimer sodium PDT + one additional topical PDT treatment after the IV PDT  -**summary**: 1 male patient with EMPD (1 lesion) age 80 years treated with ALA-PDT: 6 treatments and IV porfimer sodium PDT: 1 treatment; results: after 5 PDT treatments-CR 100%, with local recurrence at 9 months; after IV Porfimer sodium PDT-CR 100%, with local recurrence; and additional topical ALA-PDT-CR 100% at 1 year follow-up | | | | | | |
| - **Mikasa et al. (2005)** - Japan - EMPD | *Design*:  retrospective  case report  *Setting*:  single-centre, academic clinic  *Number of patients*: 2 | *Patient characteristics*  *Patient no.*: 1  *Gender*: male  *Age*: 92 years  *Length of Follow-up*: 2 months  *Lesion characteristics*  *No. of lesions*: NR  *Location of lesion(s):* penile region  *Size of lesion(s)*: NR  *History*  *Metastases*: NR  *Prior treatment and response to treatment*:   - total extirpation 15 years prior 🡪 recurrence - surgical excision deemed inappropriate (elderly pt. with decreased cardiac function) | PDT:  *Skin Prep*: NR  *Drug*: 20% 5-ALA  *Dosage*: NR  *Route of Admin*.: topical  *Time to Photoact.*: 4-6 hours  *Light Source*: excimer dye laser (PDT EDL-1), 630nm  *Light Dose*: 100 J/cm^2^ per treatment (200 J/cm^2^ total)  *Light Intensity*: NR  *Treatment Time*: NR  *No. of Treatments*: 2 | *Outcomes*:  Lesion response  -histological exam | *Outcomes*:  -2 weeks after 2 treatments: CR, erythema remitted  (elimination of tumour cells from epidermis)  -2 months after 2 treatments: no recurrence observed | 4 |
|  |  |  |  | *Adverse Events*:  NR | *Adverse Events*:  NR |  |
|  |  | *Patient characteristics*  *Patient no.*:2  *Gender*: female  *Age*: 73 years  *Length of Follow-up*: 2 months  *Lesion characteristics*  *No. of lesions*: NR  *Location of lesion(s):* right labia majora  *Size of lesion(s)*:NR  *History*  *Metastases*: NR  *Prior treatment and response to treatment*:   - none (pt. preferred to avoid surgical excision and/or radiotherapy) | PDT:  *Skin Prep*: NR  *Drug*: 20% 5-ALA  *Dosage*: NR  *Route of Admin*.: topical  *Time to Photoact.*: 4-6 hours  *Light Source*: excimer dye laser (PDT EDL-1), 630nm  *Light Dose*: 100 J/cm^2^ per treatment (500 J/cm^2^ total)  *Light Intensity*: NR  *Treatment Time*: NR  *No. of Treatments*: 5 (3 initially, 2 additional after recurrence) | *Outcomes*:  Lesion response  -histological exam | *Outcomes*:  -3 weeks after 3 treatments: CR, erythema monthstly remitted (with slight pigmentation remaining)  (elimination of tumour cells from epidermis)  -2 months after 3 treatments: recurrence  (in periphery of lesions)  -2 months after 2 additional treatments: CR  (no more recurrence) |  |
|  |  |  |  | *Adverse Events*:  NR | *Adverse Events*:  NR |  |
| *Notes:*  -2 case reports of PDT: 1 as primary treatment, 1 PDT as follow-up treatment for recurrence after excision  -**summary**: 2 patients with EMPD, mean age ± SD: 82.5 ± 9.5 years (range: 73-92), treated with PDT; results: overall-CR 2/2 (100%) | | | | | | |
| - **Tulchinsky et al. (2004)** - Israel - EMPD | *Design*:  retrospective  case report  *Setting*:  single-centre, academic clinic  *Number of patients*: 2 | *Patient characteristics*  *Patient no.*: 1  *Gender*: female  *Age*: 74 years  *Length of Follow-up*: 12 months  *Lesion characteristics*  *No. of lesions*: NR  *Location of lesion(s):* perianal  *Size of lesion(s)*: NR  *History*  *Metastases*: none  *Prior treatment and response to treatment*:   - None | PDT:  *Skin Prep*: NR  *Drug*: NR  *Dosage*: NR  *Route of Admin*.: NR  *Time to Photoact.*: NR  *Light Source*: NR  *Light Dose*: NR  *Light Intensity*: NR  *Treatment Time*: NR  *No. of Treatments*: 1 | *Outcomes*:  Lesion response  -NR | *Outcomes*:  -after 1 treatment: PR | 4 |
|  |  |  |  | *Adverse Events*:  NR | *Adverse Events*:  NR |  |
|  |  | *Patient characteristics*  *Patient no.*:2  *Gender*: female  *Age*: 49 years  *Length of Follow-up*: 81 months  *Lesion characteristics*  *No. of lesions*: NR  *Location of lesion(s):* perianal  *Size of lesion(s)*:NR  *History*  *Metastases*: none  *Prior treatment and response to treatment*:   - wide local excision and house flaps 🡪 recurrence at 81 months post-treatment | PDT:  *Skin Prep*: NR  *Drug*: NR  *Dosage*: NR  *Route of Admin*.: NR  *Time to Photoact.*: NR  *Light Source*: NR  *Light Dose*: NR  *Light Intensity*: NR  *Treatment Time*: NR  *No. of Treatments*: 2 | *Outcomes*:  Lesion response  -histological (biopsy) exam | *Outcomes*:  -after 2 treatments: PR |  |
|  |  |  |  | *Adverse Events*:  NR | *Adverse Events*:  NR |  |
| *Notes:*  -2 patients described above (treated with PDT) were part of a group of 5 patients diagnosed with Paget’s disease at a clinic (and treated with other modalities)  -2 case reports of PDT treatment for perianal Paget’s: 1 PDT as primary treatment, 1 PDT as follow-up treatment for recurrence 81 months after excision  -**summary**: 2 female patients with EMPD (2 lesions), mean age ± SD: 61.5 ± 17.7 years (range: 49-74), treated with PDT; results: after primary treatment-PR; after 2 treatments for recurrence-PR; overall-PR 2/2 (100%) | | | | | | |
| - **Zawislak et al. (2004)** - UK - EMPD | *Design*:  retrospective case report  *Setting*:  single-centre, academic clinic  *Number of patients*: 1 | *Patient characteristics*  *Patient no.*: 1  *Gender*: female  *Age*: 66 years  *Length of Follow-up*: 3 months  *Lesion characteristics*  *No. of lesions*: 1  *Location of lesion(s):* labia majora  *Size of lesion(s)*:  4cm x 3cm (12cm^2^)  *History*  *Metastases*: none  *Prior treatment and response to treatment*:   - none - pt. reluctant to undergo surgery | PDT:  *Skin Prep*: local anesthesia *Drug*: 5-ALA (patch)  *Dosage*: 38 mg/cm^2^  *Route of Admin*.: topical  *Time to Photoact.*: 5 hours  *Light Source*: incoherent red light (Paterson Lamp), 630nm  *Light Dose*: 100 J/cm^2^  *Light Intensity*: NR  *Treatment Time*: NR  *No. of Treatments*: 4 (2-6 weeks apart + 2-1 week apart) | *Outcomes*:  Lesion response  -clinical and histological (biopsy) exam | *Outcomes*:  -after 4 treatments: CR  (recurrence after 2 treatments) | 4 |
|  |  |  |  | *Adverse Events*:  -treatment-related pain reported by patient | *Adverse Events*:  -vulvar pain during first illumination but did not require analgesia (topical analgesia applied prophylactically after initial treatment) |  |
| *Notes:*  -**summary**: 1 female patient with EMPD (1 lesion) age 66 years treated with ALA-PDT: 2 treatments initially (6 weeks apart) + 2 treatments for recurrence (1 week apart); results: after 4 treatments-CR 1/1 (100%) (recurrence after first 2 treatments); follow-up 3 months after treatment | | | | | | |
| - **Zhu et al. (2004)** - China - PD | *Design*:  retrospective case series  *Setting*:  Single-centre, academic clinic  *Number of patients*: 9 | *Patient characteristics*  *Patient no.*: 1  *Gender*: NR  *Age*: NR  *Length of Follow-up*: 3-9 months  *Lesion characteristics*  *No. of lesions*:1  *Location of lesion(s):* PD (NR)  *Size of lesion(s)*: NR  *History*  *Metastases*: no  *Prior treatment and response to treatment*:   - NR | PDT:  *Skin Prep*: NR  *Drug*: HpD  *Dosage*: 5mg/kg  *Route of Admin*.: intravenous  *Time to Photoact.*: 48-72 hours  *Light Source*: HeNe laser, 632.8nm  *Light Dose*: 300 J/cm^2^  *Light Intensity*: 600mwW  *Treatment Time*: ~20min  *No. of Treatments*: 1 | *Outcomes*:  Lesion response  -clinical and histological exam (CR: complete disappearance of tumour without recurrence up to >3 months after PDT; no pathological evidence of tumour cells) | *Outcomes*:  -CR | 4 |
|  |  |  |  | *Adverse Events*:  NR | *Adverse Events*:  NR |  |
|  |  | *Patient characteristics*  *Patient no.*: 2  *Gender*: NR  *Age*: NR  *Length of Follow-up*: 3-9 months  *Lesion characteristics*  *No. of lesions*:1  *Location of lesion(s):* PD (NR)  *Size of lesion(s)*: NR  *History*  *Metastases*: no  *Prior treatment and response to treatment*:   - NR | PDT:  *Skin Prep*: NR  *Drug*: HpD  *Dosage*: 5mg/kg  *Route of Admin*.: intravenous  *Time to Photoact.*: 48-72 hours  *Light Source*: HeNe laser, 632.8nm  *Light Dose*: 300 J/cm^2^  *Light Intensity*: 600mwW  *Treatment Time*: ~20min  *No. of Treatments*: 1 | *Outcomes*:  Lesion response  -clinical and histological exam (CR: complete disappearance of tumour without recurrence up to >3 months after PDT; no pathological evidence of tumour cells) | *Outcomes*:  -CR |  |
|  |  |  |  | *Adverse Events*:  NR | *Adverse Events*:  NR |  |
|  |  | *Patient characteristics*  *Patient no.*: 3  *Gender*: NR  *Age*: NR  *Length of Follow-up*: 3-9 months  *Lesion characteristics*  *No. of lesions*:1  *Location of lesion(s):* PD (NR)  *Size of lesion(s)*: NR  *History*  *Metastases*: no  *Prior treatment and response to treatment*:   - NR | PDT:  *Skin Prep*: NR  *Drug*: HpD  *Dosage*: 5mg/kg  *Route of Admin*.: intravenous  *Time to Photoact.*: 48-72 hours  *Light Source*: HeNe laser, 632.8nm  *Light Dose*: 300 J/cm^2^  *Light Intensity*: 600mwW  *Treatment Time*: ~20min  *No. of Treatments*: 2 | *Outcomes*:  Lesion response  -clinical and histological exam (CR: complete disappearance of tumour without recurrence up to >3 months after PDT; no pathological evidence of tumour cells) | *Outcomes*:  -CR |  |
|  |  |  |  | *Adverse Events*:  NR | *Adverse Events*:  NR |  |
|  |  | *Patient characteristics*  *Patient no.*: 4  *Gender*: NR  *Age*: NR  *Length of Follow-up*: 3-9 months  *Lesion characteristics*  *No. of lesions*:1  *Location of lesion(s):* PD (NR)  *Size of lesion(s)*: NR  *History*  *Metastases*: no  *Prior treatment and response to treatment*:   - NR | PDT:  *Skin Prep*: NR  *Drug*: HpD  *Dosage*: 5mg/kg  *Route of Admin*.: intravenous  *Time to Photoact.*: 48-72 hours  *Light Source*: HeNe laser, 632.8nm  *Light Dose*: 300 J/cm^2^  *Light Intensity*: 600mwW  *Treatment Time*: ~20min  *No. of Treatments*: 2 | *Outcomes*:  Lesion response  -clinical and histological exam (CR: complete disappearance of tumour without recurrence up to >3 months after PDT; no pathological evidence of tumour cells) | *Outcomes*:  -CR |  |
|  |  |  |  | *Adverse Events*:  NR | *Adverse Events*:  NR |  |
|  |  | *Patient characteristics*  *Patient no.*: 5  *Gender*: NR  *Age*: NR  *Length of Follow-up*: 3-9 months  *Lesion characteristics*  *No. of lesions*:1  *Location of lesion(s):* PD (NR)  *Size of lesion(s)*: NR  *History*  *Metastases*: no  *Prior treatment and response to treatment*:   - NR | PDT:  *Skin Prep*: NR  *Drug*: HpD  *Dosage*: 5mg/kg  *Route of Admin*.: intravenous  *Time to Photoact.*: 48-72 hours  *Light Source*: HeNe laser, 632.8nm  *Light Dose*: 300 J/cm^2^  *Light Intensity*: 600mwW  *Treatment Time*: ~20min  *No. of Treatments*: 2 | *Outcomes*:  Lesion response  -clinical and histological exam (CR: complete disappearance of tumour without recurrence up to >3 months after PDT; no pathological evidence of tumour cells) | *Outcomes*:  -CR |  |
|  |  |  |  | *Adverse Events*:  NR | *Adverse Events*:  NR |  |
|  |  | *Patient characteristics*  *Patient no.*: 6  *Gender*: NR  *Age*: NR  *Length of Follow-up*: 3-9 months  *Lesion characteristics*  *No. of lesions*:1  *Location of lesion(s):* PD (NR)  *Size of lesion(s)*: NR  *History*  *Metastases*: no  *Prior treatment and response to treatment*:   - NR | PDT:  *Skin Prep*: NR  *Drug*: HpD  *Dosage*: 5mg/kg  *Route of Admin*.: intravenous  *Time to Photoact.*: 48-72 hours  *Light Source*: HeNe laser, 632.8nm  *Light Dose*: 300 J/cm^2^  *Light Intensity*: 600mwW  *Treatment Time*: ~20min  *No. of Treatments*: 2 | *Outcomes*:  Lesion response  -clinical and histological exam (CR: complete disappearance of tumour without recurrence up to >3 months after PDT; no pathological evidence of tumour cells) | *Outcomes*:  -CR |  |
|  |  |  |  | *Adverse Events*:  NR | *Adverse Events*:  NR |  |
|  |  | *Patient characteristics*  *Patient no.*: 7  *Gender*: NR  *Age*: NR  *Length of Follow-up*: 3-9 months  *Lesion characteristics*  *No. of lesions*:1  *Location of lesion(s):* PD (NR)  *Size of lesion(s)*: NR  *History*  *Metastases*: no  *Prior treatment and response to treatment*:   - NR | PDT:  *Skin Prep*: NR  *Drug*: HpD  *Dosage*: 5mg/kg  *Route of Admin*.: intravenous  *Time to Photoact.*: 48-72 hours  *Light Source*: HeNe laser, 632.8nm  *Light Dose*: 300 J/cm^2^  *Light Intensity*: 600mwW  *Treatment Time*: ~20min  *No. of Treatments*: 2 | *Outcomes*:  Lesion response  -clinical and histological exam (CR: complete disappearance of tumour without recurrence up to >3 months after PDT; no pathological evidence of tumour cells) | *Outcomes*:  -CR |  |
|  |  |  |  | *Adverse Events*:  NR | *Adverse Events*:  NR |  |
|  |  | *Patient characteristics*  *Patient no.*: 8  *Gender*: NR  *Age*: NR  *Length of Follow-up*: 3-9 months  *Lesion characteristics*  *No. of lesions*:1  *Location of lesion(s):* PD (NR)  *Size of lesion(s)*: NR  *History*  *Metastases*: no  *Prior treatment and response to treatment*:   - NR | PDT:  *Skin Prep*: NR  *Drug*: HpD  *Dosage*: 5mg/kg  *Route of Admin*.: intravenous  *Time to Photoact.*: 48-72 hours  *Light Source*: HeNe laser, 632.8nm  *Light Dose*: 300 J/cm^2^  *Light Intensity*: 600mwW  *Treatment Time*: ~20min  *No. of Treatments*: 2 | *Outcomes*:  Lesion response  -clinical and histological exam (CR: complete disappearance of tumour without recurrence up to >3 months after PDT; no pathological evidence of tumour cells) | *Outcomes*:  -CR |  |
|  |  |  |  | *Adverse Events*:  NR | *Adverse Events*:  NR |  |
|  |  | *Patient characteristics*  *Patient no.*: 9  *Gender*: NR  *Age*: NR  *Length of Follow-up*: 3-9 months  *Lesion characteristics*  *No. of lesions*:1  *Location of lesion(s):* PD (NR)  *Size of lesion(s)*: NR  *History*  *Metastases*: underlying adenocarcinoma  *Prior treatment and response to treatment*:   - NR | PDT:  *Skin Prep*: NR  *Drug*: HpD  *Dosage*: 5mg/kg  *Route of Admin*.: intravenous  *Time to Photoact.*: 48-72 hours  *Light Source*: HeNe laser, 632.8nm  *Light Dose*: 300 J/cm^2^  *Light Intensity*: 600mwW  *Treatment Time*: ~20min  *No. of Treatments*: 1 | *Outcomes*:  Lesion response  -clinical and histological exam (CR: complete disappearance of tumour without recurrence up to >3 months after PDT; no pathological evidence of tumour cells) | *Outcomes*:  NR* |  |
|  |  |  |  | *Adverse Events*:  NR | *Adverse Events*:  NR |  |
| *Notes:*  -9 patients described above were part of a group of 35 patients with several skin conditions at a clinic in China treated with PDT; full demographic and lesion characteristics and follow-up details not described per patient (only for general population of 35 patients)  -*outcome not reported for 1 of 9 patients; this patient was excluded from review  -**summary**: 8 patients with unspecified PD (8 lesions) – outcomes not reported for 1 patient with underlying adenocarcinoma; results: -CR 8/8 (100%) | | | | | | |
| - **Song et al. (2003)** - Korea - EMPD | *Design*:  retrospective  case series  *Setting*:  single-centre, academic clinic  *Number of patients*: 2 | *Patient characteristics*  *Patient no.*: 1  *Gender*: female  *Age*: 78 years  *Length of Follow-up*: 12 months  *Lesion characteristics*  *No. of lesions*: 2  *Location of lesion(s):*  pubis  labia major  *Size of lesion(s)*: NR  *History*  *Metastases*: NR  *Prior treatment and response to treatment*:   - None for pubis - NR for labia major | PDT:  *Skin Prep*: NR  *Drug*: 5-ALA  *Dosage*: NR  *Route of Admin*.: topical  *Time to Photoact.*: NR  *Light Source*: 600-800nm  *Light Dose*: 216 J/cm^2^  *Light Intensity*: 120 mW/cm^2^  *Treatment Time*: NR  *No. of Treatments*: 4 (per lesion) | *Outcomes*:  Lesion response  -histological (biopsy) exam | *Outcomes*:  -after 4 treatments: 2/2 (100%) CR | 4 |
|  |  |  |  | *Adverse Events*:  NR | *Adverse Events*:  NR |  |
|  |  | *Patient characteristics*  *Patient no.*:2  *Gender*: male  *Age*: 51 years  *Length of Follow-up*: 12 months  *Lesion characteristics*  *No. of lesions*: 4  *Location of lesion(s):*  pubis  R penile base  R penile shaft  R scrotum  *Size of lesion(s)*:NR  *History*  *Metastases*: NR  *Prior treatment and response to treatment*:   - NR | PDT:  *Skin Prep*: NR  *Drug*: 5-ALA  *Dosage*: NR  *Route of Admin*.: topical  *Time to Photoact.*: NR  *Light Source*: 600-800nm  *Light Dose*: R penile shaft 144 J/cm^2^; other lesions 216 J/cm^2^  *Light Intensity*: 120 mW/cm^2^  *Treatment Time*: NR  *No. of Treatments*: 4 (per lesion) | *Outcomes*:  Lesion response  -histological (biopsy) exam | *Outcomes*:  -after 4 treatments: 3/4 (75%) CR; 1/4 (25%) PR (R penile base 🡪 excised) |  |
|  |  |  |  | *Adverse Events*:  NR | *Adverse Events*:  NR |  |
| *Notes:*  -only abstract and table available in English (NR refers to not reported in the abstract or table)  -2 patients described above (treated with PDT) were part of a group of 6 patients diagnosed with various skin conditions  -**summary**: 2 patients with EMPD (6 lesions), mean age ± SD: 64.5 ± 19.1 years (range: 51-78), treated with PDT; results: after 4 treatments 5/6 (83%) CR; 1/6 (17%) PR | | | | | | |
| - **Xu et al. (2002)** - China - EMPD, MPD | *Design*:  retrospective case series  *Setting*:  Single-centre, academic clinic  *Number of patients*: 10 (EMPD 8, MPD 2) | *Patient characteristics*  *Patient no.*: 1  *Gender*: male  *Age*: NR  *Length of Follow-up*: NR  *Lesion characteristics*  *No. of lesions*:1  *Location of lesion(s):* EM (NR)  *Size of lesion(s)*: NR  *History*  *Metastases*: NR  *Prior treatment and response to treatment*:   - NR | PDT:  *Skin Prep*: NR  *Drug*: 5-ALA (10% solution or 20% cream)  *Dosage*: NR  *Route of Admin*.: topical (wet dressing)  *Time to Photoact.*: 3-4 hours  *Light Source*: HeNe laser, 632.8nm  *Light Dose*: 72-100 J/cm^2^ (per irradiation)  *Light Intensity*: NR  *Treatment Time*: ~30min (longer for larger tumours)  *No. of Treatments*: NR (but performed at 2 week intervals) | *Outcomes*:  Lesion response  -histological exam | *Outcomes*:  -CR | 4 |
|  |  |  |  | *Adverse Events*:  Not reported by disease/case specific reports | *Adverse Events*:  NR |  |
|  |  | *Patient characteristics*  *Patient no.*: 2  *Gender*: male  *Age*: NR  *Length of Follow-up*: NR  *Lesion characteristics*  *No. of lesions*:1  *Location of lesion(s):* EM (NR)  *Size of lesion(s)*: NR  *History*  *Metastases*: NR  *Prior treatment and response to treatment*:   - NR | (same as above) | *Outcomes*:  Lesion response  -histological exam | *Outcomes*:  -CR |  |
|  |  |  |  | *Adverse Events*:  Not reported by disease/case specific reports | *Adverse Events*:  NR |  |
|  |  | *Patient characteristics*  *Patient no.*: 3  *Gender*: male  *Age*: NR  *Length of Follow-up*: NR  *Lesion characteristics*  *No. of lesions*:1  *Location of lesion(s):* EM (NR)  *Size of lesion(s)*: NR  *History*  *Metastases*: NR  *Prior treatment and response to treatment*:   - NR | (same as above) | *Outcomes*:  Lesion response  -histological exam | *Outcomes*:  -CR |  |
|  |  |  |  | *Adverse Events*:  Not reported by disease/case specific reports | *Adverse Events*:  NR |  |
|  |  | *Patient characteristics*  *Patient no.*: 4  *Gender*: male  *Age*: NR  *Length of Follow-up*: NR  *Lesion characteristics*  *No. of lesions*:1  *Location of lesion(s):* EM (NR)  *Size of lesion(s)*: NR  *History*  *Metastases*: NR  *Prior treatment and response to treatment*:   - NR | (same as above) | *Outcomes*:  Lesion response  -histological exam | *Outcomes*:  -CR |  |
|  |  |  |  | *Adverse Events*:  Not reported by disease/case specific reports | *Adverse Events*:  NR |  |
|  |  | *Patient characteristics*  *Patient no.*: 5  *Gender*: male  *Age*: NR  *Length of Follow-up*: NR  *Lesion characteristics*  *No. of lesions*:1  *Location of lesion(s):* EM (NR)  *Size of lesion(s)*: NR  *History*  *Metastases*: NR  *Prior treatment and response to treatment*:   - NR | (same as above) | *Outcomes*:  Lesion response  -histological exam | *Outcomes*:  -PR (>50% of tissue died) |  |
|  |  |  |  | *Adverse Events*:  Not reported by disease/case specific reports | *Adverse Events*:  NR |  |
|  |  | *Patient characteristics*  *Patient no.*: 6  *Gender*: male  *Age*: NR  *Length of Follow-up*: NR  *Lesion characteristics*  *No. of lesions*:1  *Location of lesion(s):* EM (NR)  *Size of lesion(s)*: NR  *History*  *Metastases*: NR  *Prior treatment and response to treatment*:   - NR | (same as above) | *Outcomes*:  Lesion response  -histological exam | *Outcomes*:  -PR |  |
|  |  |  |  | *Adverse Events*:  Not reported by disease/case specific reports | *Adverse Events*:  NR |  |
|  |  | *Patient characteristics*  *Patient no.*:7  *Gender*: male  *Age*: NR  *Length of Follow-up*: NR  *Lesion characteristics*  *No. of lesions*:1  *Location of lesion(s):* EM (NR)  *Size of lesion(s)*: NR  *History*  *Metastases*: NR  *Prior treatment and response to treatment*:   - NR | (same as above) | *Outcomes*:  Lesion response  -histological exam | *Outcomes*:  -PR |  |
|  |  |  |  | *Adverse Events*:  Not reported by disease/case specific reports | *Adverse Events*:  NR |  |
|  |  | *Patient characteristics*  *Patient no.*: 8  *Gender*: male  *Age*: NR  *Length of Follow-up*: NR  *Lesion characteristics*  *No. of lesions*:1  *Location of lesion(s):* EM (NR)  *Size of lesion(s)*: NR  *History*  *Metastases*: NR  *Prior treatment and response to treatment*:   - NR | (same as above) | *Outcomes*:  Lesion response  -histological exam | *Outcomes*:  -PR |  |
|  |  |  |  | *Adverse Events*:  Not reported by disease/case specific reports | *Adverse Events*:  NR |  |
|  |  | *Patient characteristics*  *Patient no.*: 9  *Gender*: female  *Age*: NR  *Length of Follow-up*: NR  *Lesion characteristics*  *No. of lesions*:1  *Location of lesion(s):* MPD (nipple)  *Size of lesion(s)*: NR  *History*  *Metastases*: NR  *Prior treatment and response to treatment*:   - NR | (same as above) | *Outcomes*:  Lesion response  -histological exam | *Outcomes*:  -CR  (“complete reaction” after combination PDT + surgery) |  |
|  |  |  |  | *Adverse Events*:  Not reported by disease/case specific reports | *Adverse Events*:  NR |  |
|  |  | *Patient characteristics*  *Patient no.*: 10  *Gender*: female  *Age*: NR  *Length of Follow-up*: NR  *Lesion characteristics*  *No. of lesions*:1  *Location of lesion(s):* MPD (nipple)  *Size of lesion(s)*: NR  *History*  *Metastases*: NR  *Prior treatment and response to treatment*:   - NR | (same as above) | *Outcomes*:  Lesion response  - histological exam | *Outcomes*:  -PR  (“complete reaction” after combination PDT + surgery) |  |
|  |  |  |  | *Adverse Events*:  Not reported by disease/case specific reports | *Adverse Events*:  NR |  |
| *Notes:*  -10 patients described above were part of a group of 88 patients with several skin conditions at a clinic in China treated with PDT; full demographic and lesion characteristics, specific intervention characteristics and follow-up details not described per patient (only for general population of 88 patients treated with PDT at clinic)  -**summary**: 10 patients with EMPD (8 male) and MPD-nipple (2 female) (10 lesions), median age: 61.9 years (range: 50-84 years); results: EMPD-CR 4/8 (50%), PR 4/8 (50%); MPD-CR1/2 (50%), PR 1/2 (50%); overall: CR 5/10 (50%), PR 5/10 (50%). Follow-up for this group of patients ranged from 3-36 months (EMPD) and 18-36 months (MPD)  -the authors note “complete reaction” for the 2 cases of MPD nipple after combined ALA-PDT and surgery  -although AE’s not reported per patient, authors note some for all 88 patients overall: no uncomfortable sensation during ALA application, prickling occasionally during irradiation, erythema around lesion 1-3 days after treatment, crust formation and peeling within 1 week after treatment | | | | | | |
| - **Chang et al. (2001)** - Korea - EMPD | *Design*:  retrospective  case series  *Setting*:  single-centre,  academic clinic  *Number of patients*:7 | *Patient characteristics*  *Patient no.*: 1  *Gender*: male  *Age*: 68 years  *Length of Follow-up*: 18 months  *Lesion characteristics*  *No. of lesions*: 1  *Location of lesion(s):*  penis/scrotum  *Size of lesion(s)*:  2.7x3.4cm  *History*  *Metastases*: NR  *Prior treatment and response to treatment*:   - NR | PDT:  Treatment 1  *Skin Prep*: NR  *Drug*: 20% 5-ALA  *Dosage*: NR  *Route of Admin*.: topical  *Time to Photoact.*: NR  *Light Source*: Wood’s lamp  *Light Dose*: 125-200 J/cm^2^  *Light Intensity*: 50-100 mW/cm^2^  *Treatment Time*: NR  *No. of Treatments*: NR  Treatment 2  *Skin Prep*: NR  *Drug*: 0.5mg/mL HpD  *Dosage*: NR  *Route of Admin*.: intralesional injection  *Time to Photoact.*: NR  *Light Source*: Wood’s lamp  *Light Dose*: 125-200 J/cm^2^  *Light Intensity*: 50-100 mW/cm^2^  *Treatment Time*: NR  *No. of Treatments*: NR  Treatment 3  *Skin Prep*: NR  *Drug*: HpD  *Dosage*: 2mg/kg  *Route of Admin*.: intravenous  *Time to Photoact.*: NR  *Light Source*: short-arc xenon lamp (Auctilux®, Australia)  *Light Dose*: 125-200 J/cm^2^  *Light Intensity*: 50-100 mW/cm^2^  *Treatment Time*: NR  *No. of Treatments*: NR | *Outcomes*:  Lesion response  -NR | *Outcomes*:  -PR | 4 |
|  |  |  |  | *Adverse Events*:  -NR | *Adverse Events*:  -“side effects were minimal” |  |
|  |  | *Patient characteristics*  *Patient no.*: 2  *Gender*: male  *Age*: 56 years  *Length of Follow-up*: 12 months  *Lesion characteristics*  *No. of lesions*: 1  *Location of lesion(s):*  scrotum  *Size of lesion(s)*:  3.3x3.0cm  *History*  *Metastases*: NR  *Prior treatment and response to treatment*:   - NR | (same as above) | *Outcomes*:  Lesion response  -NR | *Outcomes*:  -PR |  |
|  |  |  |  | *Adverse Events*:  -NR | *Adverse Events*:  -“side effects were minimal” |  |
|  |  | *Patient characteristics*  *Patient no.*: 3  *Gender*: male  *Age*: 54 years  *Length of Follow-up*: 24 months  *Lesion characteristics*  *No. of lesions*: 1  *Location of lesion(s):*  penis/scrotum  *Size of lesion(s)*:  4.5x5.0  *History*  *Metastases*: NR  *Prior treatment and response to treatment*:   - NR | (same as above) | *Outcomes*:  Lesion response  -NR | *Outcomes*:  -PR |  |
|  |  |  |  | *Adverse Events*:  -NR | *Adverse Events*:  -“side effects were minimal” |  |
|  |  | *Patient characteristics*  *Patient no.*: 4  *Gender*: male  *Age*: 73 years  *Length of Follow-up*: 18 months  *Lesion characteristics*  *No. of lesions*: 1  *Location of lesion(s):*  penis/scrotum  *Size of lesion(s)*:  3.1x3.9cm  *History*  *Metastases*: NR  *Prior treatment and response to treatment*:   - NR | (same as above) | *Outcomes*:  Lesion response  -NR | *Outcomes*:  -PR |  |
|  |  |  |  | *Adverse Events*:  -NR | *Adverse Events*:  -“side effects were minimal” |  |
|  |  | *Patient characteristics*  *Patient no.*: 5  *Gender*: male  *Age*: 53 years  *Length of Follow-up*: 22 months  *Lesion characteristics*  *No. of lesions*: 1  *Location of lesion(s):*  scrotum  *Size of lesion(s)*:  2.7x3.0cm  *History*  *Metastases*: NR  *Prior treatment and response to treatment*:   - NR | (same as above) | *Outcomes*:  Lesion response  -NR | *Outcomes*:  -PR |  |
|  |  |  |  | *Adverse Events*:  -NR | *Adverse Events*:  -“side effects were minimal” |  |
|  |  | *Patient characteristics*  *Patient no.*: 6  *Gender*: female  *Age*: 58 years  *Length of Follow-up*: 18 months  *Lesion characteristics*  *No. of lesions*: 2  *Location of lesion(s):*  R vulva  L vulva  *Size of lesion(s)*:  2.5x5.8cm  3.1x6.3cm  *History*  *Metastases*: NR  *Prior treatment and response to treatment*:   - NR | (same as above) | *Outcomes*:  Lesion response  -NR | *Outcomes*:  -PR |  |
|  |  |  |  | *Adverse Events*:  -NR | *Adverse Events*:  -“side effects were minimal” |  |
|  |  | *Patient characteristics*  *Patient no.*: 7  *Gender*: male  *Age*: 50 years  *Length of Follow-up*: 20 months  *Lesion characteristics*  *No. of lesions*: 1  *Location of lesion(s):*  penis/scrotum  *Size of lesion(s)*:  2.8x3.2cm  *History*  *Metastases*: NR  *Prior treatment and response to treatment*:   - NR | (same as above) | *Outcomes*:  Lesion response  -NR | *Outcomes*:  -PR |  |
|  |  |  |  | *Adverse Events*:  -NR | *Adverse Events*:  -“side effects were minimal” |  |
| *Notes:*  -only abstract and table available in English (NR refers to not reported in the abstract or table)  -**summary**: 7 patients with 8 EMPD lesions, mean age ± SD: 58.9 ± 8.5 years (range: 50-73), treated with PDT; results: PR 8/8 (100%) | | | | | | |
| - **Runfola et al. (2000)** - USA - EMPD | *Design*:  retrospective  case report  *Setting*:  single-centre,  academic clinic  *Number of patients*: 1 | *Patient characteristics*  *Patient no.*: 1  *Gender*: male  *Age*: 79 years  *Length of Follow-up*: 6 years  *Lesion characteristics*  *No. of lesions*: 1  *Location of lesion(s):* perianal  *Size of lesion(s)*: 7cm  *History*  *Metastases*: NR (nodal status was negative for all patients)  *Prior treatment and response to treatment*:   - excision and skin graft 🡪 recurrence | PDT:  *Skin Prep*: NR  *Drug*: Porfimer sodium (Photofrin®)  *Dosage*: 1mg/kg  *Route of Admin*.: intravenous  *Time to Photoact.*: 24-48 hours  *Light Source*: 630nm argon pumped dye laser (Coherent Innova 100+, Coherent, Inc., Santa Clara, CA)  *Light Dose*: 210 J/cm^2^  *Light Intensity*: 150 mW/cm^2^  *Treatment Time*: NR  *No. of Treatments*: 1 | *Outcomes*:  Lesion response  - clinical assessment and histological (biopsy) exam if observed presence of lesion | *Outcomes*:  -CR  -recurrence at 4 years post-treatment  -unrelated death at 6 years | 4 |
|  |  |  |  | *Adverse Events*:  -perianal erythema: clinical examination 36-48 hours post treatment  -pain: patient reported | *Adverse Events*:  -perianal erythema and blister formation observed within 48hrs post-treatment  -fecal diversion was not required  -pain: analgesic management by either oral acetaminophen with codeine 4-6hrs and/or IV morphine (pain is reported in 4 to 5 pts., 2 pts. required re-admission for pain management – not specified which patients) |  |
| *Notes:*  -1 patient described above was part of a group of 5 patients with several skin conditions at a clinic in USA treated with PDT; full patient-specific adverse event data was not available  -**summary**: 1 male patient with EMPD (1 lesion) age 79 years treated with 1 courses of IV Porfimer sodium-PDT, for local recurrence after conventional treatment failure; results: after 1 treatment: perianal-CR 1/1 (100%), with recurrence at 4 years post treatment and death of unrelated disease at 6 years follow-up | | | | | | |
| - **Henta et al. (1999)** - Japan - EMPD | *Design*:  retrospective  case report  *Setting*:  single-centre,  academic clinic  *Number of patients*: 1 | *Patient characteristics*  *Patient no.*: 1  *Gender*: female  *Age*: 74 years  *Length of Follow-up*: NR  *Lesion characteristics*  *No. of lesions*: 1  *Location of lesion(s):* vulva  *Size of lesion(s)*:  20cm x 25cm (500cm^2^)  *History*  *Metastases*: yes (lymphatic bilateral inguinal and pulmonary regions)  *Prior treatment and response to treatment*:   - electron beam irradiation & oral etoposide 100mg chemotherapy - inguinal masses disappeared; vulva lesion reduced to 60% of original size | PDT:  Treatment 1  *Skin Prep*: NR  *Drug*: 20% δ-ALA  *Dosage*: NR  *Route of Admin*.: topical  *Time to Photoact.*: 4 hours  *Light Source*: red filtered polychromatic halogen (1000-W) light, 600-700nm  *Light Dose*: 500 J/cm^2^  *Light Intensity*: 200 mW/cm^2^  *Treatment Time*: NR  *No. of Treatments*: 1  Treatment 2  *Skin Prep*: topical lidocaine  *Drug*: 10% δ-ALA in saline  *Dosage*: 10ml  *Route of Admin*.: intralesional injection  *Time to Photoact.*: 4 hours  *Light Source*: red filtered polychromatic halogen (1000-W) light, 600-700 nm.  *Light Dose*: 500 J/cm^2^  *Light Intensity*: 200 mW/cm^2^  *Treatment Time*: NR  *No. of Treatments*: 10 (2 weeks apart) | *Outcomes*:  Lesion response  - clinical and histological (biopsy) exam | *Outcomes*:  -after 1 topical (PR) & 10 IV PDT treatments: CR (“histologically disease free” to 7mm depth) | 4 |
|  |  |  |  | QoL  -reported by author | -authors report treatment “improved the patient’s QoL” |  |
|  |  |  |  | *Adverse Events*:  -pain and burning sensation reported by patient | *Adverse Events*:  -pain treated with good effect using lidocaine topical cream  -burning sensation well tolerated by patient |  |
| *Notes:*  -**summary**: 1 patient with metastatic, advanced, extensive and inoperable EMPD (1 lesion with deep penetration) age 74 years treated with δ-ALA-PDT after chemo-radiotherapy treatment of metastasis; results: after 11 treatments-CR 1/1 (100%) patient (“histologically disease free”) | | | | | | |
| - **Wang et al. (1991)** - China - EMPD, PD | *Design*:  retrospective  case series  *Setting*:  single-centre, academic clinic  *Number of patients*: 7 (EMPD 4, PD 3) | *Patient characteristics*  *Patient no.*: 1  *Gender*: NR  *Age*: NR  *Length of Follow-up*: NR  *Lesion characteristics*  *No. of lesions*: 1  *Location of lesion(s):* EM (NR)  *Size of lesion(s)*: NR  *History*  *Metastases*: NR  *Prior treatment and response to treatment*:   - none | PDT:  *Skin Prep*: NR  *Drug*: Hematoporphyrin derivative (HpD) 50mg/10ml/ampule  *Dosage*: 3-5mg/kg body weight  *Route of Admin*.: intravenous  *Time to Photoact.*: 48 hours  *Light Source*: HeNe laser or argon-pumped dye laser or flash-lamp pumped dye laser  *Light Dose*: NR  *Light Intensity*: HeNe: 50-180 mW/cm^2^, argon: 200 mW/cm^2^, flash-lamp: 400 mW/cm^2^  *Treatment Time*: NR  *No. of Treatments*: NR (at 2 day intervals) | *Outcomes*:  Lesion response  -grade of response:  Excellent: complete remission  Good: 70% remission  Fair: 20-70% remission  Poor: No effect or < 20% remission with rapid regrowth | *Outcomes*:  -excellent | 4 |
|  |  |  |  | *Adverse Events*:  -blood and urine testing; liver function tests | *Adverse Events*:  -normal test results |  |
|  |  | *Patient characteristics*  *Patient no.*: 2  *Gender*: NR  *Age*: NR  *Length of Follow-up*: NR  *Lesion characteristics*  *No. of lesions*: 1  *Location of lesion(s):* EM (NR)  *Size of lesion(s)*: NR  *History*  *Metastases*: NR  *Prior treatment and response to treatment*:   - none | (same as above) | *Outcomes*:  Lesion response  -grade of response:  Excellent: complete remission  Good: 70% remission  Fair: 20-70% remission  Poor: No effect or < 20% remission with rapid regrowth | *Outcomes*:  -excellent |  |
|  |  |  |  | *Adverse Events*:  -blood and urine testing; liver function tests | *Adverse Events*:  -normal test results |  |
|  |  | *Patient characteristics*  *Patient no.*: 3  *Gender*: NR  *Age*: NR  *Length of Follow-up*: NR  *Lesion characteristics*  *No. of lesions*: 1  *Location of lesion(s):* EM (NR)  *Size of lesion(s)*: NR  *History*  *Metastases*: NR  *Prior treatment and response to treatment*:   - none | (same as above) | *Outcomes*:  Lesion response  -grade of response:  Excellent: complete remission  Good: 70% remission  Fair: 20-70% remission  Poor: No effect or < 20% remission with rapid regrowth | *Outcomes*:  -good |  |
|  |  |  |  | *Adverse Events*:  -blood and urine testing; liver function tests | *Adverse Events*:  -normal test results |  |
|  |  | *Patient characteristics*  *Patient no.*: 4  *Gender*: NR  *Age*: NR  *Length of Follow-up*: NR  *Lesion characteristics*  *No. of lesions*: 1  *Location of lesion(s):* EM (NR)  *Size of lesion(s)*: NR  *History*  *Metastases*: NR  *Prior treatment and response to treatment*:   - none | (same as above) | *Outcomes*:  Lesion response  -grade of response:  Excellent: complete remission  Good: 70% remission  Fair: 20-70% remission  Poor: No effect or < 20% remission with rapid regrowth | *Outcomes*:  -good |  |
|  |  |  |  | *Adverse Events*:  -blood and urine testing; liver function tests | *Adverse Events*:  -normal test results |  |
|  |  | *Patient characteristics*  *Patient no.*: 5  *Gender*: NR  *Age*: NR  *Length of Follow-up*: NR  *Lesion characteristics*  *No. of lesions*: 1  *Location of lesion(s):* PD (NR)  *Size of lesion(s)*: NR  *History*  *Metastases*: NR  *Prior treatment and response to treatment*:   - none | (same as above) | *Outcomes*:  Lesion response  -grade of response:  Excellent: complete remission  Good: 70% remission  Fair: 20-70% remission  Poor: No effect or < 20% remission with rapid regrowth | *Outcomes*:  -excellent |  |
|  |  |  |  | *Adverse Events*:  -blood and urine testing; liver function tests | *Adverse Events*:  -normal test results |  |
|  |  | *Patient characteristics*  *Patient no.*: 6  *Gender*: NR  *Age*: NR  *Length of Follow-up*: NR  *Lesion characteristics*  *No. of lesions*: 1  *Location of lesion(s):* PD (NR)  *Size of lesion(s)*: NR  *History*  *Metastases*: NR  *Prior treatment and response to treatment*:   - none | (same as above) | *Outcomes*:  Lesion response  -grade of response:  Excellent: complete remission  Good: 70% remission  Fair: 20-70% remission  Poor: No effect or < 20% remission with rapid regrowth | *Outcomes*:  -excellent |  |
|  |  |  |  | *Adverse Events*:  -blood and urine testing; liver function tests | *Adverse Events*:  -normal test results |  |
|  |  | *Patient characteristics*  *Patient no.*: 7  *Gender*: NR  *Age*: NR  *Length of Follow-up*: NR  *Lesion characteristics*  *No. of lesions*: 1  *Location of lesion(s):* PD (NR)  *Size of lesion(s)*: NR  *History*  *Metastases*: NR  *Prior treatment and response to treatment*:   - none | (same as above) | *Outcomes*:  Lesion response  -grade of response:  Excellent: complete remission  Good: 70% remission  Fair: 20-70% remission  Poor: No effect or < 20% remission with rapid regrowth | *Outcomes*:  -fair |  |
|  |  |  |  | *Adverse Events*:  -blood and urine testing; liver function tests | *Adverse Events*:  -normal test results |  |
| *Notes:*  -7 patients described above were part of a group of 50 patients with several skin conditions at a clinic in China treated with PDT; full demographic and lesion characteristics, specific intervention characteristics and follow-up details not described per patient  -**summary**: 7 patients with EMPD (4) and PD (3) lesions treated with PDT as primary treatment; results: EMPD-Excellent 2/4 (50%), Good 2/4 (50%); PD- Excellent 2/3 (67%), Fair 1/3 (33%); overall-Excellent 4/7 (57%), Good 2/7 (29%), Fair 1/7 (14%)  -excellent = complete remission, good = 70% remission, fair = 20-70% remission, poor = no effect or <20% remission followed by rapid growth  -although AE’s not reported per patient, authors note some for all 50 patients overall: slight phototoxic reactions seen in 2/50 patients who did not avoid sunlight after treatment, 1/50 patients with hyperplastic scar formation after PDT + CO2 laser | | | | | | |
| - **Kubota et al. (1986)** - Japan - EMPD | *Design*:  retrospective  case series  *Setting*:  single-centre,  academic clinic  *Number of patients*: 1 | *Patient characteristics*  *Patient no.*: 1  *Gender*: female  *Age*: 77 years  *Length of Follow-up*: 6 months  *Lesion characteristics*  *No. of lesions*: 1  *Location of lesion(s):* vulva  *Size of lesion(s)*:  NR  *History*  *Metastases*: NR  *Prior treatment and response to treatment*:   - NR | PDT:  *Skin Prep*: NR  *Drug*: Photofrin  *Dosage*: 2.5mg/kg  *Route of Admin*.: intravenous  *Time to Photoact.*: NR  *Light Source*: laser  *Light Dose*: NR  *Light Intensity*: 150-200mW/cm^2^  *Treatment Time*: 20-40min  *No. of Treatments*: NR | *Outcomes*:  Lesion response  -clinical and histological | *Outcomes*:  -NC | 4 |
|  |  |  |  | *Adverse Events*:  -NR | *Adverse Events*:  -NR |  |
| *Notes:*  -only abstract and table available in English (NR refers to not reported in the abstract or table)  -1 patient described above (treated with PDT) was part of a group of 14 patients with several skin conditions treated with PDT at a clinic in Japan  -**summary**: 1 patients with EMPD, 77 years, treated with PDT; results: 0/1 (0%) CR | | | | | | |
| NR = not reported  N/A = not applicable  CR = complete response, PR = partial response, MR = minimal response, NC = no change in lesion before and after treatment (i.e., no response) | | | | | | |
