# Supplementary material for: Effectiveness of photodynamic therapy for mammary and extra-mammary Paget's disease: a state of the science review
Source: BMC Dermatol. 2011 Jun 15;11:13. doi: 10.1186/1471-5945-11-13 (PMC3141658; doi:10.1186/1471-5945-11-13)
Supplement: Additional file 3 — Studies of photodynamic therapy (PDT) for Extramammary Paget's disease: lesion response. Details of individual patient and lesion characteristics, type and number of PDT treatments, lesion response outcomes and lengths of follow-up are provided in additional file 3. [file 1471-5945-11-13-S3.DOC]

### Additional file 3 - Studies of photodynamic therapy (PDT) for Extramammary Paget's disease: lesion response

| **Study** | **Patient ID** | **Age (years)** | **Gender** | **Prior treatment*** | **Lesion location** | **Type of PDT** | **Number of treatments** | **Response** | **Length of follow-up** (months)** |
| --- | --- | --- | --- | --- | --- | --- | --- | --- | --- |
| **Prospective case series***** | | | | | | | | | |
| Raspagliesi et al. (2006) | 1 | 55 | female | surgical and laser excision | vulva | topical MAL-PDT | 3 | Complete Response | 5 |
|  |  |  |  | surgical and laser excision | perianal | topical MAL-PDT | 3 | No Response | 5 |
|  | 2 | 60 | female | laser excision | vulva | topical MAL-PDT | 3 | No Response | 4 |
|  | 3 | 57 | female | surgical and laser excision | vulva | topical MAL-PDT | 3 | Complete Response | 4 |
|  |  |  |  | surgical and laser excision | perianal | topical MAL-PDT | 3 | Complete Response | 4 |
|  | 4 | 67 | female | surgical excision | vulva | topical MAL-PDT | 3 | Complete Response | 4 |
|  |  |  |  | surgical excision | perianal | topical MAL-PDT | 3 | Complete Response | 4 |
|  | 5 | 59 | female | surgical and laser excision | vulva | topical MAL-PDT | 3 | Complete Response | 2 |
|  | 6 | 69 | female | surgical and laser excision | vulva | topical MAL-PDT | 3 | Complete Response | 1 |
|  | 7 | 75 | female | laser excision | vulva | topical MAL-PDT | 3 | Partial Response | 1 |
|  |  |  |  | laser excision | axilla | topical MAL-PDT | 3 | Complete Response | 1 |
| **Retrospective case reports/case series** | | | | | | | | | |
| Housel et al. (2010) and Shieh et al. (2002) | 8 | 75 | male | surgical excision | axilla | topical ALA-PDT | 1 | Partial Response | 19 |
|  | 9 | 50 | male | none | groin | 1^st^ 4 treatments: topical ALA-PDT; last treatment: intravenous Porfimer sodium-PDT | 5 | Complete Response | 96 |
|  | 10 | 61 | male | laser excision | pubis | topical ALA-PDT | 3 | Partial Response | 20 |
|  |  |  |  | laser excision | penis | topical ALA-PDT | 3 | Partial Response | 20 |
|  |  |  |  | laser excision | scrotum | topical ALA-PDT | 3 | Partial Response | 20 |
|  | 11 | 65 | male | laser excision | pubis | topical ALA-PDT | 1 | Complete Response | 9 |
|  |  |  |  | laser excision | buttock | topical ALA-PDT | 1 | Complete Response | 9 |
|  |  |  |  | laser excision | pubis | topical ALA-PDT | 1 | Complete Response | 9 |
|  |  |  |  | laser excision | scrotum | topical ALA-PDT | 2 | Complete Response | 6 |
|  |  |  |  | laser excision | scrotum | topical ALA-PDT | 2 | Complete Response | 6 |
|  |  |  |  | none | scrotum | topical ALA-PDT | 2 | Complete Response | 6 |
|  | 12 | 72 | male | MMS | scrotum | topical ALA-PDT | 2 | Partial Response | 6 |
|  |  |  |  | MMS | scrotum | topical ALA-PDT | 2 | Partial Response | 6 |
|  |  |  |  | none | penis | topical ALA-PDT | 1 | Complete Response | 88 |
|  |  |  |  | none | penis | topical ALA-PDT | 3 | Partial Response | 4 |
|  |  |  |  | none | penis | topical ALA-PDT | 1 | Complete Response | 47 |
|  | 13 | 78 | female | surgical excision and topical ALA-PDT | perianal | intravenous Porfimer sodium-PDT | 1 | Complete Response | 68 |
|  |  |  |  | topical ALA-PDT | perianal | intravenous Porfimer sodium-PDT | 1 | Partial Response | 48 |
|  |  |  |  | topical ALA-PDT | perianal | intravenous Porfimer sodium-PDT | 1 | Partial Response | 48 |
|  | 14 | 80 | female | none | perianal | intravenous Porfimer-Sodium PDT | 1 | Complete Response | 12 |
|  |  |  |  | none | buttock | intravenous Porfimer sodium-PDT | 1 | Complete Response | 12 |
|  |  |  |  | none | perianal | intravenous Porfimer sodium-PDT | 1 | Complete Response | 12 |
|  | 15 | 52 | female | surgical and laser excision | perianal | intravenous Porfimer sodium-PDT | 1 | Complete Response | 62 |
|  |  |  |  | surgical and laser excision | perianal | intravenous Porfimer sodium-PDT | 1 | Complete Response | 62 |
| Thaler et al. (2010) | 16 | 69 | male | ? | perianal | topical ALA-PDT | 4 | Partial Response | ? |
| Andretta-Tanaka et al. (2009) | 17 | 68 | male | topical imiquimod | axilla | topical MAL-PDT | 8 | Partial Response | 12 |
|  | 18 | 76 | male | none | scrotum | topical MAL-PDT | 3 | Complete Response | 6 |
|  | 19 | 73 | female | none | vulva | topical MAL-PDT | 3 | Partial Response | 6 |
|  | 20 | 67 | female | none | vulva | topical MAL-PDT | 3 | Partial Response | 6 |
| Fukui et al. (2009) | 21 | 81 | male | topical ALA-PDT | penis | topical ALA-PDT | 6 | Complete Response | 18 |
|  | 22 | 84 | female | topical ALA-PDT | vulva | topical ALA-PDT | 9 | Complete Response | 17 |
|  | 23 | 66 | female | surgical excision | vulva | topical ALA-PDT | 9 | Complete Response | 15 |
|  |  |  |  | surgical excision | perianal | topical ALA-PDT | 9 | Complete Response | 15 |
|  | 24 | 76 | female | surgical excision | vulva | topical ALA-PDT | ? | Complete Response | 12 |
|  |  |  |  | surgical excision | extra-urethra | topical ALA-PDT | ? | Complete Response | 12 |
|  | 25 | 83 | female | surgical excision | vulva | topical ALA-PDT | ? | Complete Response | 3 |
|  |  |  |  | surgical excision | vulva | topical ALA-PDT | ? | Complete Response | 3 |
| Li et al. (2009) | 26 | 75 | female | none | perianal | 1^st^ 2 treatments: topical ALA-PDT; 3^rd^ treatment: topical ALA-PDT + intravenous HpD^a^-PDT | 3 | Complete Response | 7 |
| Wang et al. (2008) | 27 | 66 | male | cryotherapy | penis/scrotum | topical ALA-PDT | 3 | Partial Response | 12 |
|  | 28 | ? | ? | ? | unspecified EM | topical ALA-PDT | ? | Partial Response | 12 |
|  | 29 | ? | ? | ? | unspecified EM | topical ALA-PDT | ? | Partial Response | 12 |
| Liu et al. (2007) | 31 | ? | male | ? | scrotum | intravenous Porfimer sodium-PDT | 3 | ?^b^ | 3 |
|  | 32 | ? | male | ? | penis | intravenous Porfimer sodium-PDT | 3 | ?^b^ | 3 |
|  | 33 | ? | male | ? | crissum | intravenous Porfimer sodium-PDT | 3 | ?^b^ | 3 |
|  | 34 | ? | female | ? | pubis | intravenous Porfimer sodium-PDT | 3 | ?^b^ | 3 |
|  | 35 | ? | female | ? | vulva | intravenous Porfimer sodium-PDT | 3 | ?^b^ | 3 |
| T’Kint & Roseeuw (2006) | 36 | 64 | female | MMS | vulva | topical MAL-PDT | 4 | Complete Response | 14 |
|  |  |  |  | MMS | perianal | topical MAL-PDT | 4 | Complete Response | 14 |
| Kim et al. (2005) | 37 | 54 | male | ? | scrotum | combined PDT and surgical excision^c^ | ? | Complete Response | ? |
|  | 38 | 69 | male | ? | penis/scrotum | combined PDT and surgical excision^c^ | ? | Complete Response | ? |
|  | 39 | 56 | male | ? | scrotum | combined PDT and surgical excision^c^ | ? | Complete Response | ? |
|  | 40 | 73 | male | ? | penis/scrotum | combined PDT and surgical excision^c^ | ? | Complete Response | ? |
|  | 41 | 58 | female | ? | vulva | combined PDT and surgical excision^c^ | ? | Complete Response | ? |
|  | 42 | 54 | male | ? | penis/scrotum | combined PDT and surgical excision^c^ | ? | Complete Response | ? |
|  | 43 | 50 | male | ? | penis/scrotum | combined PDT and surgical excision^c^ | ? | Complete Response | ? |
| Madan et al. (2005) | 44 | 80 | male | none | groin/scrotum | 1^st^ 5 and last treatments: topical ALA-PDT; 6^th^ treatment: intravenous Porfimer sodium-PDT | 7 | Complete Response | 12 |
| Mikasa et al. (2005) | 45 | 92 | male | surgical excision | penis | topical ALA-PDT | 2 | Complete Response | 2 |
|  | 46 | 73 | female | none | vulva | topical ALA-PDT | 5 | Complete Response | 2 |
| Tulchinsky et al. (2004) | 47 | 74 | female | none | perianal | ? | 1 | Partial Response | 12 |
|  | 48 | 49 | female | surgical excision | perianal | ? | 2 | Partial Response | ? |
| Zawislak et al. (2004) | 49 | 66 | female | none | vulva | topical ALA-PDT | 4 | Complete Response | 3 |
| Zhu et al. (2004) | 50 | ? | ? | ? | unspecified PD | intravenous HpD^a^-PDT | 1 | Complete Response | ? |
|  | 51 | ? | ? | ? | unspecified PD | intravenous HpD^a^-PDT | 1 | Complete Response | ? |
|  | 52 | ? | ? | ? | unspecified PD | intravenous HpD^a^-PDT | 2 | Complete Response | ? |
|  | 53 | ? | ? | ? | unspecified PD | intravenous HpD^a^-PDT | 2 | Complete Response | ? |
|  | 54 | ? | ? | ? | unspecified PD | intravenous HpD^a^-PDT | 2 | Complete Response | ? |
|  | 55 | ? | ? | ? | unspecified PD | intravenous HpD^a^-PDT | 2 | Complete Response | ? |
|  | 56 | ? | ? | ? | unspecified PD | intravenous HpD^a^-PDT | 2 | Complete Response | ? |
|  | 57 | ? | ? | ? | unspecified PD | intravenous HpD^a^-PDT | 2 | Complete Response | ? |
| Song et al. (2003) | 58 | 78 | female | none | pubis | topical ALA-PDT | 4 | Complete Response | 12 |
|  |  |  |  | NR | vulva | topical ALA-PDT | 4 | Complete Response | 12 |
|  | 59 | 51 | male | NR | pubis | topical ALA-PDT | 4 | Complete Response | 12 |
|  |  |  |  | NR | penis | topical ALA-PDT | 4 | Partial Response | 12 |
|  |  |  |  | NR | penis | topical ALA-PDT | 4 | Complete Response | 12 |
|  |  |  |  | NR | scrotum | topical ALA-PDT | 4 | Complete Response | 12 |
| Xu et al. (2002) | 60 | ? | male | ? | unspecified EM | topical ALA-PDT | ? | Complete Response | ? |
|  | 61 | ? | male | ? | unspecified EM | topical ALA-PDT | ? | Complete Response | ? |
|  | 62 | ? | male | ? | unspecified EM | topical ALA-PDT | ? | Complete Response | ? |
|  | 63 | ? | male | ? | unspecified EM | topical ALA-PDT | ? | Complete Response | ? |
|  | 64 | ? | male | ? | unspecified EM | topical ALA-PDT | ? | Partial Response | ? |
|  | 65 | ? | male | ? | unspecified EM | topical ALA-PDT | ? | Partial Response | ? |
|  | 66 | ? | male | ? | unspecified EM | topical ALA-PDT | ? | Partial Response | ? |
|  | 67 | ? | male | ? | unspecified EM | topical ALA-PDT | ? | Partial Response | ? |
| Chang et al. (2001) | 70 | 68 | male | ? | penis/scrotum | combined topical ALA-PDT, intralesional injection HpD^a^-PDT and intravenous HpD^a^-PDT | ? | Partial Response | 18 |
|  | 71 | 56 | male | ? | scrotum | combined topical ALA-PDT, intralesional injection HpD^a^-PDT and intravenous HpD^a^-PDT | ? | Partial Response | 12 |
|  | 72 | 54 | male | ? | penis/scrotum | combined topical ALA-PDT, intralesional injection HpD^a^-PDT and intravenous HpD^a^-PDT | ? | Partial Response | 24 |
|  | 73 | 73 | male | ? | penis/scrotum | combined topical ALA-PDT, intralesional injection HpD^a^-PDT and intravenous HpD^a^-PDT | ? | Partial Response | 18 |
|  | 74 | 53 | male | ? | scrotum | combined topical ALA-PDT, intralesional injection HpD^a^-PDT and intravenous HpD^a^-PDT | ? | Partial Response | 22 |
|  | 75 | 58 | female | ? | L vulva | combined topical ALA-PDT, intralesional injection HpD^a^-PDT and intravenous HpD^a^-PDT | ? | Partial Response | 18 |
|  |  |  |  | ? | R vulva | combined topical ALA-PDT, intralesional injection HpD^a^-PDT and intravenous HpD^a^-PDT | ? | Partial Response | 18 |
|  | 76 | 50 | male | ? | penis/scrotum | combined topical ALA-PDT, intralesional injection HpD^a^-PDT and intravenous HpD^a^-PDT | ? | Partial Response | 20 |
| Runfola et al. (2000) | 77 | 79 | male | surgical excision | perianal | intravenous Porfimer sodium-PDT | 1 | Partial Response | 48 |
| Henta et al. (1999) | 78 | 74 | female | electron beam irradiation and chemotherapy | vulva | 1^st^ treatment: topical ALA-PDT; 2^nd^ treatment: intralesional injection ALA-PDT | 10 | Complete Response | ? |
| Wang et al. (1991) | 79 | ? | ? | none | unspecified EM | intravenous HpD^a^-PDT | ? | Complete Response | ? |
|  | 80 | ? | ? | none | unspecified EM | intravenous HpD^a^-PDT | ? | Complete Response | ? |
|  | 81 | ? | ? | none | unspecified EM | intravenous HpD^a^-PDT | ? | Partial Response | ? |
|  | 82 | ? | ? | none | unspecified EM | intravenous HpD^a^-PDT | ? | Partial Response | ? |
|  | 83 | ? | ? | none | unspecified PD | intravenous HpD^a^-PDT | ? | Complete Response | ? |
|  | 84 | ? | ? | none | unspecified PD | intravenous HpD^a^-PDT | ? | Complete Response | ? |
|  | 85 | ? | ? | none | unspecified PD | intravenous HpD^a^-PDT | ? | Partial Response | ? |
| Kubota et al. (1986) | 86 | 77 | female | ? | vulva | intravenous Porfimer sodium-PDT | ? | No Response | 6 |
| * history of prior treatment for Paget’s disease  ** length of follow-up after last treatment  *** an additional prospective case series study was included in the review (Li et al. (2010)) but not included in this table as outcomes were not reported by patient  ^a^ unspecified hematoporphyrin derivative (HpD) used as photosensitizing agent  ^b^ Liu et al. (2007) did not report outcome data by patient; overall outcomes: 1/5 lesions complete response, 3/5 partial response, 1/5 minimal response  ^c^ order of treatment not reported (i.e. whether surgery or PDT was performed first)  ? = not specified, ALA = 5-aminolevulinic acid, MAL = methyl aminolevulinate, MMS = Mohs Micrographic surgery, HpD = hematoporphyrin derivative | | | | | | | | | |
