# Supplementary material for: Effectiveness of photodynamic therapy for mammary and extra-mammary Paget's disease: a state of the science review
Source: BMC Dermatol. 2011 Jun 15;11:13. doi: 10.1186/1471-5945-11-13 (PMC3141658; doi:10.1186/1471-5945-11-13)
Supplement: Additional file 4 — Studies of photodynamic therapy (PDT) for Mammary Paget's disease: lesion response. Details of individual patient and lesion characteristics, type and number of PDT treatments, lesion response outcomes and lengths of follow-up are provided in additional file 4. [file 1471-5945-11-13-S4.DOC]

### Additional file 4 - Studies of photodynamic therapy (PDT) for Mammary Paget's disease: lesion response

| **Study** | **Patient ID** | **Age (years)** | **Gender** | **Prior treatment*** | **Lesion location** | **Type of PDT** | **Number of treatments** | **Response** | **Length of follow-up* (months)** |
| --- | --- | --- | --- | --- | --- | --- | --- | --- | --- |
| **Retrospective case reports/case series** | | | | | | | | | |
| Wang et al. (2008) | 30 | 28 | female | radical mastectomy | mammary | topical ALA-PDT | 2 | Complete Response | 12 |
| Xu et al. (2002) | 68 | ? | female | ? | mammary | combined topical ALA-PDT and surgical excision^a^ | ? | Complete Response | ? |
|  | 69 | ? | female | ? | mammary | combined topical ALA-PDT and surgical excision^a^ | ? | Complete Response | ? |
| ^a^ authors do not report order of treatment (i.e. whether surgery or PDT was performed first) | | | | | | | | | |
